# Peptide targeting of lysophosphatidylinositol-sensing GPR55 for osteoclastogenesis tuning

**DOI:** 10.1186/s12964-021-00727-w

**Published:** 2021-04-26

**Authors:** Maria Giovanna Mosca, Maria Mangini, Stefania Cioffi, Pasquale Barba, Stefania Mariggiò

**Affiliations:** 1 Institute of Protein Biochemistry, National Research Council, Naples, Italy; 2 Institute of Genetics and Biophysics, National Research Council, Naples, Italy; 3 Present Address: Institute of Biochemistry and Cell Biology, National Research Council, Naples, Italy

**Keywords:** G-protein-coupled receptor (GPCR), GPR55, Lysophosphatidylinositol (LPI), Peptidic binder, Osteoclastogenesis

## Abstract

**Background:**

The G-protein-coupled receptor GPR55 has been implicated in multiple biological activities, which has fuelled interest in its functional targeting. Its controversial pharmacology and often species-dependent regulation have impacted upon the potential translation of preclinical data involving GPR55.

**Results:**

With the aim to identify novel GPR55 regulators, we have investigated lysophosphatidylinositol (LPI)-induced GPR55-mediated signal transduction. The expression system for wild-type and mutated GPR55 was HeLa cells silenced for their endogenous receptor by stable expression of a short-hairpin RNA specific for *GPR55* 5′-UTR, which allowed definition of the requirement of GPR55 Lys^80^ for LPI-induced MAPK activation and receptor internalisation. In RAW264.7 macrophages, GPR55 pathways were investigated by *Gpr55* silencing using small-interfering RNAs, which demonstrated that LPI increased intracellular Ca^2+^ levels and induced actin filopodium formation through GPR55 activation. Furthermore, the LPI/GPR55 axis was shown to have an active role in osteoclastogenesis of precursor RAW264.7 cells induced by ‘receptor-activator of nuclear factor kappa-β ligand’ (RANKL). Indeed, this differentiation into mature osteoclasts was associated with a 14-fold increase in *Gpr55* mRNA levels. Moreover, GPR55 silencing and antagonism impaired RANKL-induced transcription of the osteoclastogenesis markers: ‘nuclear factor of activated T-cells, cytoplasmic 1′, matrix metalloproteinase-9, cathepsin-K, tartrate-resistant acid phosphatase, and the calcitonin receptor, as evaluated by real-time PCR. Phage display was previously used to identify peptides that bind to GPR55. Here, the GPR55-specific peptide-P1 strongly inhibited osteoclast maturation of RAW264.7 macrophages, confirming its activity as a blocker of GPR55-mediated functions. Although osteoclast syncytium formation was not affected by pharmacological regulation of GPR55, osteoclast activity was dependent on GPR55 signalling, as shown with resorption assays on bone slices, where LPI stimulated and GPR55 antagonists inhibited bone erosion.

**Conclusions:**

Our data indicate that GPR55 represents a target for development of novel therapeutic approaches for treatment of pathological conditions caused by osteoclast-exacerbated bone degradation, such as in osteoporosis or during establishment of bone metastases.

**Video abstract**

**Supplementary Information:**

The online version contains supplementary material available at 10.1186/s12964-021-00727-w.

## Background

G-protein-coupled receptors (GPCRs) are attractive targets for drug discovery as they regulate a vast array of physiological processes and have accessible ‘druggable’ sites [1]. Furthermore, their pharmacological manipulation represents an already validated approach for treatment of numerous diseases. To date about 34% of drugs on the market are directed towards GPCRs, and the targeting of these receptors was proposed to be promising also for cancer treatment [2]. An analysis of all GPCR drugs in clinical trials highlighted the trends across all molecule types, particularly in favour of biologics, allosteric modulators, and ligands with biased signalling [1].

GPR55 belongs to the δ group of rhodopsin-like (Class A) GPCRs [3, 4], and the distribution of its mRNA expression has been detailed in different organisms; however, information regarding the expression levels of the GPR55 protein is still lacking [5]. GPR55 is widely expressed in several mammalian tissues, including breast, adipose tissue, testes and spleen [6], and several regions of the brain [7]. GPR55 has been implicated in different pathophysiological conditions, such as vascular functions [8], bone turnover [9, 10], neuropathic/inflammatory pain [11, 12], motor coordination [13], central nervous system disorders [14, 15], metabolic dysfunction [5, 16], immune dysregulation [17] and alterations that drive malignant cell growth [18, 19].

For a long time, GPR55 was classified as a cannabinoid receptor [20], as after its discovery and cloning [21], different studies demonstrated that endogenous, plant and synthetic cannabinoids can bind to and activate GPR55 [22]. However, GPR55 is phylogenetically distinct from the traditional cannabinoid receptors, and human GPR55 shows only 13.5% and 14.4% homology with human CB_1_ and CB_2_, respectively [20]. Subsequent in-vitro screening led to identification of new GPR55 ligands that are unrelated to the cannabinoid system [23–28]. Furthermore, the International Union of Basic and Clinical Pharmacology (IUPHAR) still classifies GPR55 as an orphan receptor, with lysophosphatidylinositol (LPI), and in particular 2-arachidonoylglycerolphosphoinositol [29], proposed as the natural/endogenous ligand [30]. Indeed, LPI has been shown to bind to and activate GPR55 in vitro [20, 31], although whether this activation occurs in vivo is still under investigation [30].

The LPIs are a group of lysolipids that are characterised by a glycerol backbone with a single fatty acid substitution, which is linked to the* myo*-inositol molecule by a phosphodiester bond [32]. The acyl chain can be different depending on its position on the glycerol backbone, its length, and the number of its unsaturated bonds. LPI can be produced from the membrane component phosphatidylinositol by the catalytic activity of phospholipases A_1_ or A_2_, which catalyse the hydrolysis of the acyl chains at the *sn*-1 or *sn*-2 positions, respectively, on the glycerol backbone [31]. LPI has also been implicated in different pathophysiological processes, including cell migration [33] and proliferation [34], neuropathic pain [35], bone remodelling [9] and cancer progression [36, 37].

Whyte and collaborators addressed the physiological relevance of the LPI/GPR55 axis in bone metabolism [9]. Indeed, a *Gpr55*-knockout mouse model showed a significant increase in volume and thickness of the trabecular bone, and an excess of non-resorbed cartilage. They demonstrated that this bone phenotype was consequent to increased numbers of morphologically inactive osteoclasts [9]. However, little is known about the mechanism of action of GPR55 in the osteoclastogenesis process.

On the basis of the relevance of GPR55 in several biological functions, many efforts have been dedicated to its targeting. However, these have been challenged by the difficulties arisen from GPR55 complicated pharmacology and its often species-dependent regulation. This has made difficult to understand the potential for its translation to the clinic [5].

Here we have dissected out LPI-activated GPR55 signalling, which highlights the requirement of GPR55 Lys^80^ for LPI recognition, and the relevance of the LPI/GPR55 axis in the osteoclastogenesis process and in osteoclast bone resorption. Furthermore, we have characterised a peptide that specifically recognises and binds to GPR55, as both the human and murine receptor. This provides an example of a valuable tool with potential application to targeted and combination therapies in bone pathologies with exacerbated osteoclast activity.

## Materials and methods

### Materials

Dulbecco’s modified Eagle’s medium (DMEM), minimum essential medium (MEM), foetal bovine serum (FBS), non-essential amino acids, Hanks balanced salt solution with calcium and magnesium (HBSS^++^), and phosphate-buffered saline (PBS) were from Gibco (Life Technologies Italia, Italy). Penicillin–streptomycin, L-glutamine, non-fat milk, bovine serum albumin (BSA), fatty-acid-free (faf)-BSA, Tween-20, MEM Eagle alpha-modified (α-MEM), Hoechst, CID16020046, and L-α-lysophosphatidylinositol sodium salt from soybean were from Sigma-Aldrich (St. Louis, MO, USA). Purified synthetic 1-palmitoyl-2-hydroxy-sn-glycero-3-phosphoinositol (16:0 LPI), 1-stearoyl-2-hydroxy-sn-glycero-3-phosphoinositol (18:0 LPI), 1-oleoyl-2-hydroxy-sn-glycero-3-phospho-(1′-myo-inositol) (18:1 LPI), 1-arachidonoyl-2-hydroxy-sn-glycero-3-phosphoinositol (20:4 LPI) were from Avanti Polar Lipids, Inc. (Alabaster, AL, USA). ML-191, ML-184, and O1918 were from Cayman Chemical (Ann Arbor, MI, USA). Cannabidiol (CBD) was from Tocris Bioscience (Bristol, UK). Mowiol 4–88 and puromycin were from Calbiochem (San Diego, CA, USA). Lipofectamine 2000, Lipofectamine LTX with Plus reagent, Alexa488-tagged anti-mouse antibody, and Alexa546-labelled phalloidin, were from Invitrogen (Carlsbad, CA, USA). The mouse monoclonal anti-HA (16B12) antibody was from Covance (Princeton, NJ, USA). Paraformaldehyde was from Electron Microscopy Sciences (Hatfield, PA, USA). ‘Receptor-activator of nuclear factor kappa-β ligand’ (RANKL) was from Peprotech (London, UK). Ionomycin, was from Santa Cruz Biotechnology (San Diego, CA, USA). All of the synthetic peptides were from Caslo ApS (Lyngby, Denmark). Based on the sequence of peptide-P1 (CKKNSPTLC), both a scrambled peptide (Scr; KCLTSNCPK) with the same amino-acid composition as peptide-P1 but a different primary sequence, and an irrelevant peptide (Irr_P; CGGNGPGLC) that included mutations to all of the polar amino acids of peptide-P1, were designed. All of the peptides were cyclised using an intramolecular disulphide bond between the two cysteine residues [38]. The fluorescent peptides were obtained by conjugation at the N-terminus with fluorescein-isothiocyanate (FITC) with an aminohexanoic acid linker. All other reagents were obtained at the highest purities available from Merck Life Science (Milano, Italy).

### Site-directed mutagenesis

The construct of haemagglutinin (HA)-tagged human GPR55 in pcDNA3 (HA-GPR55) was a gift from Prof. K. Mackie, Indiana University, Bloomington, IN, USA [39], while the construct ss-3 × HA-GPR55 in pcDNA3.1 (ssGPR55) with a triple HA tag at the N-terminus and an optimised signal sequence (ss, derived from amino acids 1–33 of the human growth hormone: MATGSPTSLLLAFGLLCLPWLQEGSARDPPVAT) for efficient surface expression was from Prof. A. Irving, Dundee University, UK [40]. For both constructs, mutations were introduced by site-directed mutagenesis using QuickChange kits (Stratagene, La Jolla, CA, USA), according to the manufacturer instructions. The primers for the K80A mutation were 5′-CTCTCCCTCCCATTCGCGATGGTCCTGTCCCAG-3′ and 5′-CTGGGACAGGACCATCGCGAATGGGAGGGAGAG-3′ (Tm, 70.6 °C), and for Q87A were 5′-GTCCTGTCCCAGGTAGCGTCCCCCTTCCCGTCC-3′ and 5′-GGACGGGAAGGGGGACGCTACCTGGGACAGGAC-3′ (Tm, 73.1 °C).

### RNA extraction and real-time PCR

Total RNA was extracted using RNeasy isolation kits, cDNAs were obtained using QuantiTect Reverse Transcription kits, and real-time PCRs were performed with QuantiTect SYBR Green PCR kits (all from Qiagen, Hilden, Germany), according to the manufacturer instructions. The primers used for the real-time PCRs and their annealing temperatures are listed in the Additional file 1: Table S1. Human hypoxanthine phosphoribosyltransferase 1 (*HPRT1*) or murine *β*_*2*_*-microglobulin* were followed as housekeeping genes. The real-time PCR programme consisted of an initial 15 min at 95 °C, and then 45 cycles as follows: 94 °C for 15 s, annealing temperature of each primer for 30 s, and 72 °C for 30 s. The real-time PCR machine used was a LightCycler 480 Instrument II (Roche, Indianapolis, IN, USA).

### Cell culture

HEK293T cells were bought in 2012 from American Type Culture Collection (293 T/17; ATCC catalogue number: CRL-11268), and were grown in monolayers in DMEM supplemented with 10% FBS, 2 mM L-glutamine, 100 U/mL penicillin and 100 µg/mL streptomycin.

HeLa cells were received from Dr. Corda's laboratory (Institute of Biochemistry and Cell Biology, CNR of Naples) that bought them in 2006 from the European Collection of Cell Culture (ECACC catalogue number: 93021013). HeLa cells were maintained in MEM with 10% FBS, 2 mM L-glutamine, 100 U/mL penicillin, 100 µg/mL streptomycin, and non-essential amino acids.

The RAW264.7 murine monocyte/macrophages were bought in 2003 from ATCC (catalogue number: TIB-71), and were cultured in DMEM with 10% heat-inactivated (30 min at 55 °C) FBS, 2 mM L-glutamine, 100 U/mL penicillin and 100 µg/mL streptomycin.

All of the cells were tested free of mycoplasma, and were grown in a humidified atmosphere of 5% CO_2_ at 37 °C.

### Transfection and RNA interference

For GPR55 overexpression, HEK293T cells were plated in their growth medium without antibiotics at 2.6 × 10^5^ cells/well in 12-well plates, and 24 h later, the cells were transfected with 1 µg cDNA/well using Lipofectamine 2000, according to the manufacturer instructions. The pcDNA3 empty vector or that coding for human HA-GPR55 wild-type or its mutants HA-GPR55-K80A and HA-GPR55-Q87A were used.

HeLa cells were plated at 1.5 × 10^5^ cells/well in six-well plates in their growth medium without antibiotics. Twenty-four hours later, the cells were transfected with 2.5 µg/well pcDNA3.1, or the mutants ssGPR55-K80A, ssGPR55-Q87A, or 1.25 µg/well (complemented with 1.25 µg/well empty vector) ssGPR55, using Lipofectamine 2000, according to the manufacturer instructions. The different cDNA amounts were necessary to reach equivalent plasma-membrane expression of the receptors, as the mutants were expressed at lower levels, as verified by FACS analyses, and in line with previous reports [41].

For stable interference of *GPR55*, HeLa cells were plated at 1.5 × 10^5^ cells/well in six-well plates in growth medium without antibiotics, and 24 h later, the cells were transfected with 1.7 µg/well OmicsLink short hairpin (sh)RNA expression clone CSHCTR001-CU6 (shCTRL) or clone HSH022476-3-CU6 (shGPR55) from GeneCopoeia (Rockville, MD, USA), using Lipofectamine 2000, according the manufacturer instructions. Forty-eight hours after transfection, HeLa clones stably expressing shRNAs were selected in growth medium containing 0.3 µg/mL puromycin. The efficiency of interference was monitored by real-time PCR using the primers listed in the Additional file 1: Table S1. *HPRT1* was followed as a housekeeping gene.

For transient interference of *Gpr55*, RAW264.7 cells were plated at 6 × 10^5^ cells/well in six-well plates in growth medium without antibiotics. Twenty-four hours later, the cells were transfected with 250 pmol/well non-targeting small-interfering (si)RNAs (si-NT; siGENOME siRNA Pool #2; D-001206-14; Dharmacon, Chicago, IL, USA) or *Gpr55*-specific siRNAs (si-GPR55; siGENOME mouse GPR55 SMART pool; M-043590-01; Dharmacon) using Lipofectamine LTX and Plus Reagent, according to the manufacturer instructions. Twenty-four hours later, the cells were plated for the different assays or for RNA extraction. The efficiency of interference was monitored by real-time PCR after 72 h of interference, using the primers listed in the Additional file 1: Table S1. *β*_*2*_*-microglobulin* was followed as a housekeeping gene. Interfered samples that showed < 40% reduction in *Gpr55* mRNA were not analysed further.

### Cell stimulation

Twenty-four hours after transfection (HEK293T, HeLa cells or clones) or 72 h after interference (RAW264.7 cells), the cells were washed twice with HBSS^++^, serum deprived (HEK293T cells for 4 h in DMEM; HeLa cells and clones for 2 h in MEM plus 2 mM glutamine and 25 mM HEPES; RAW264.7 cells for 2 h in DMEM), washed once again with HBSS^++^, incubated in stimulation buffer (HBSS^++^ with 10 mM HEPES, 0.4% faf-BSA for HEK293T cells; HBSS^++^ with 25 mM HEPES and 0.01% faf-BSA for HeLa cells, clones, and RAW264.7 cells) in the absence or presence of stimuli, at 37 °C for the indicated times. Incubations were terminated by washing the cells twice with cold HBSS^++^, and the analyses were performed as reported below.

### Western blotting

Cell lysates were obtained by scraping the cells into phospho-lysis buffer: 50 mM Tris–HCl, pH 7.5, 100 mM NaCl, 5 mM EDTA, 1% Triton X-100, 50 mM NaF, 40 mM β-glycerophosphate, 200 µM sodium orthovanadate, plus protease and phosphatase inhibitors (Roche). Following gentle homogenisation by 20 passages through a 26-gauge needle, the lysates were centrifuged at 10,000×*g* for 5 min at 4 °C, and the supernatants were collected.

Protein lysates were subjected to SDS-PAGE, and after electrophoresis, the proteins were transferred to a nitrocellulose membrane (PerkinElmer Life Science, Boston, MA, USA). For immunoblotting, the membranes were blocked with 5% non-fat milk in TBS (10 mM Tris–HCl, pH 7.4, 10 mM NaCl) plus 0.1% Tween-20 (T-TBS) for 30 min at room temperature, and incubated with primary antibodies in T-TBS plus 3% BSA for 2 h at room temperature, or overnight at 4 °C. The membranes were washed twice in T-TBS for 7 min, and then incubated with secondary antibodies conjugated to horseradish peroxidase (1:5,000) (Calbiochem, San Diego, CA, USA) in T-TBS with 5% non-fat milk for 30 min at room temperature. The membranes were then washed twice with T-TBS and once with TBS for 5 min, and the signals were detected by ECL (Amersham Pharmacia, Piscataway, NJ, USA). The rabbit anti-phospho AKT (Ser473), anti-phospho p38 (Thr180/Tyr182), anti-phospho p42/44 (Thr202/Tyr204), anti-p38 (all at dilution 1:1000) were from Cell Signaling Technology (Danvers, MA, USA). The rabbit anti-AKT (B-1), and anti-p42/44 (ERK1; K-23) were from Santa Cruz Biotechnology.

### Ca^2+^ assay

After 48 h of siRNA treatments, the RAW264.7 cells were detached with 600 μM EDTA in PBS, and plated at a density of 8 × 10^4^ cells/well in 96-well plates. Seventy-two hours from the interference, the cells underwent Ca^2+^ measurements using Fluo4-NW Calcium Assay kits (Invitrogen), according to the manufacturer instructions. Interfered cells were washed twice with HBSS^++^, incubated with 50 μL loading buffer (0.01% faf-BSA, 20 mM HEPES in HBSS^++^, 5 mM probenecid, and 2 × Fluo4-NW) for 45 min at 37 °C. All the subsequent incubation steps were performed at 37 °C within the Fluoroskan Ascent FL (Thermo Fisher Scientific, Waltham, MA USA) and the fluorescence recorded with an Ex 485/ Em 520 every 3 s. The baseline fluorescence was monitored for 5 min, then 50 μL assay buffer (0.01% faf-BSA, 20 mM HEPES in HBSS^++^) was added without or with 10 μM 16:0 LPI, and fluorescence was recorded for a further 5 min. Subsequently, the cells were stimulated with addition of 2 μL ionomycin (1 μM final concentration, for F_max_), 2 μL EGTA (6 mM final concentration, F_min_) and 2 μL CaCl_2_ (8 mM final concentration) in sequence, and the fluorescence recorded for 2 min for each stimulus. The intracellular Ca^2+^ concentrations were calculated according to Eq. (1):

1 $$\left[ {{\text{Ca}}^{2 + } } \right]_{{{\text{free}}}} = {\text{ K}}_{{\text{d}}} \left[ {{\text{F}} - {\text{F}}_{{{\text{min}}}} ]  /} \right[{\text{F}}_{\max } - {\text{F}}],$$

using the Fluo-4 K_d_ of 345 nM.

### Cytoskeleton analysis

Twenty-four hours after plating the RAW264.7 cells at a density of 1.2 × 10^6^ cells/well in six-well plates, or 2.5 × 10^5^ cells/well in 24-well plates on coverslips, the cells were serum deprived for 2 h and then stimulated while adhered, with LPI in the assay buffer (0.1% faf-BSA, 20 mM HEPES in HBSS^++^). Stimulation was blocked by two washes with HBSS^++^, and cells on coverslips were processed for immunofluorescence (see below), while the cells in the six-well plates were scraped into cytoskeleton buffer (10 mM 4-morpholineethanesulfonic acid, 150 mM NaCl, 5 mM EGTA, 5 mM MgCl_2_, 5 mM glucose), for FACS analysis. For the latter, an equal volume of fixation solution (1% Triton X-100, 0.5% glutaraldehyde, in cytoskeleton buffer) was added to the cell suspension and left for 2 min at room temperature. The cells were then washed twice (5 min each) with cytoskeleton buffer, fixed again for 15 min with 1% glutaraldehyde in cytoskeleton buffer at room temperature, further washed three times (10 min each) with cytoskeleton buffer, treated with 500 mg/mL sodium borohydride for 10 min on ice, and washed three times (10 min each) with cytoskeleton buffer. Finally, the fixed cells were stained with 33 nM Alexa546-labelled phalloidin for 1 h at room temperature, washed three times (10 min each) with cytoskeleton buffer, suspended in PBS with 3% BSA, and analysed by FACS (FACSCalibur or FACSAria III; Becton Dickinson, Franklin Lakes, NJ).

### Immunofluorescence microscopy

For actin staining, the cells were rinsed with HBSS^++^, fixed in 4% (w/v) paraformaldehyde for 10 min at room temperature, and permeabilised with blocking solution (50 mM ammonium chloride, 0.5% BSA, 0.1% saponin, 0.02% NaN_3_, in PBS), for 30 min at room temperature. The cells were stained for a further 1 h at room temperature with 33 nM Alexa488-labelled phalloidin for filamentous actin visualisation, and 2 μg/mL Hoechst for nucleus staining, with all of the reagents diluted in blocking solution. Then the cells were washed three times with PBS plus 0.02% Tween-20, and the coverslips were mounted with Mowiol 4–88 and examined under confocal microscopy (LSM 510; Zeiss, Oberkochen, Germany). The cytoskeleton underwent blinded morphological scoring for filopodium formation (200 cells per sample), 63 × objective, as: absence, 0; partial response, 1; full response, 2 (see also [42]). This provided a maximum score of 400, with the data given as percentages of each response *versus* the respective control.

For the evaluation of osteoclast syncytium formation, the nuclei of multinucleated cells were counted in a blinded manner using a 63 × objective, moving across the coverslip in the vertical and horizontal directions. No evident differences in cell numbers, as a consequence of cell toxicity or changes in proliferation rates, were observed for the differentiated cells treated in the absence or presence of the GPR55 agonists/antagonists.

### GPR55 quantification by FACS

Twenty-four hours after transfection, the HEK293T cells were detached with PBS plus 1 mM EDTA, centrifuged at 300×*g* for 5 min at 4 °C, incubated in blocking buffer (5% BSA, 5% FCS, in PBS) for 30 min on ice, and then centrifuged at 300×*g* for 5 min at 4 °C. All of the subsequent steps were on ice with cold PBS plus 3% BSA. The cells were stained with a murine anti-HA antibody (1:1000) for 1 h, washed three times, further incubated in the dark with an Alexa488-tagged anti-mouse antibody (1:800) for 30 min, washed three times, suspended in PBS plus 3% BSA, and analysed by FACS.

For the GPR55-internalisation assay, after stimulation, the HeLa cells were washed twice with cold HBSS^++^, stained while adhered with the monoclonal anti-HA antibody (1:1000) in PBS plus 3% BSA for 1 h on ice, washed three times with cold PBS, incubated with the Alexa488-tagged anti-mouse antibody (1:800) in PBS plus 3% BSA for 45 min on ice. After two washes with cold PBS and a final wash with PBS at room temperature, the cells were incubated 5 min at 37 °C with PBS plus 2 mM EDTA, and detached by scraping. The collected cells were centrifuged at 300×*g*, suspended in PBS plus 3% BSA, and analysed by FACS.

### Peptide binding to RAW264.7 cells

Wild-type or 48-h-interfered RAW264.7 cells were plated at a density of 1.2 × 10^6^ cells/well in six-well plates, and the following day they were used for on-plate-binding assays, or for RNA extraction. Before peptide addition, the cells were washed twice with HBSS^++^, and then incubated without or with 40 µg/mL FITC-P1 or FITC-Scr for the indicated times at 37 °C in HBSS^++^ plus 0.01% faf-BSA. The incubations were stopped by three washes with PBS, detached by scraping with PBS plus 2 mM EDTA, and then suspended in PBS plus 3% BSA. Fluorescence intensity was evaluated by FACS, and reported as means of cell-associated fluorescence increases compared to cells incubated in absence of peptides.

To evaluate peptide-P1 specificity towards murine GPR55, RAW264.7 cells were *Gpr55*-interfered as above, but the duplexes were previously mixed with the double pmol amount of siGLO Red transfection indicator (Dharmacon). Under these conditions, a 40% reduction in *Gpr55* mRNA levels was measured in the total population of si-GPR55-treated versus si-NT-treated cells, and 30% of both populations were siGLO-positive, as verified by FACS analysis. Peptide binding was evaluated only towards siGLO-positive cells, which were assumed to have a higher proportion of siRNA-treated cells compared to the total population. FITC-fluorescence intensity was evaluated by FACS, and reported as means of cell-associated fluorescence increases compared to cells incubated in absence of peptides.

### Osteoclastogenesis in-vitro assay

For the osteoclastogenesis *in-vitro* assay, RAW264.7 cells were plated in differentiation medium (α-MEM with 10% heat-inactivated FBS, 2 mM L-glutamine, 100 U/mL penicillin, and 100 ug/mL streptomycin) at a density of 5 × 10^3^ cells/well in 24-well plates on coverslips for morphological analysis, or at 2 × 10^4^ cells/well in six-well plates for RNA extraction. Twenty-four hours later and every 48 h, the medium was replaced and the cells were treated with 15–30 ng/mL RANKL with DMSO and/or PBS as carriers, or with GPR55 antagonists/ agonists (0.5 µM ML-191, 30 µM O1918, 0.5 µM CBD, 0.5 μM CID16020046 1 µM soybean LPI, 1 μM ML-184), or with the peptides (150 nM peptide-P1; or the irrelevant peptide, Irr_P). Twenty-four hours after the last addition, the cells were fixed with 4% paraformaldehyde for morphological analyses or harvested for RNA extraction (see above).

### Osteoclast functional assay

RAW264.7 cells were plated in differentiation medium at a density of 1250 cells/100 µL on 0.2-mm-thick bovine cortical bone slices (BoneSlices.com, Jelling, Denmark) in 96-well plates. Six hours later, 100 µL differentiation medium with RANKL (15 ng/mL, final concentration) was added, in the presence of a carrier or in combination with the GPR55 modulators. The media with the different compounds were replaced every 48 h, for a total of 7 days. At the end of this period, the cells were detached with a 10% bleach solution, and the resorption excavations were visualised by using Toluidine Blue staining (Sigma-Aldrich). Images of the resorption areas were obtained under the microscope (SW380T; Swift Optical Instruments, Inc., TX, USA) using the 10 × objective, and acquired with a digital camera (Swiftcam SC1003; Swift Optical Instruments, Inc.). The total eroded surface underwent blinded quantification using ImageJ (NIH), and was subdivided into pit and trench surfaces. Pits were characterised as round excavations with well-defined edges where the ratio between length and width was < 2.0. Trenches were defined as elongated excavations with well-defined edges whose length was at least twice the wide, and with clear signs of continued resorption. The prevalence of trenches was calculated as a proportion (%) of the trench surface relative to the total eroded surface [43].

### Statistical analysis

Statistical analysis was performed with the GraphPad Prism software (GraphPad Software, Inc. La Jolla, CA, USA). Comparisons between groups were performed using Student’s *t*-test and Analysis of Variance (ANOVA) with 95% confidence interval. *p* < 0.05 was considered statistically significant.

## Results

### LPI-dependent GPR55-mediated signal transduction

The activation of GPR55 by both cannabinoid [16, 22] and non-cannabinoid [28, 31, 44] ligands still fuels research into its pharmacology. The initial definition of LPI-activated GPR55-mediated signal transduction was addressed in GPR55-overexpressing HEK293 cells, where phosphorylation of extracellular signal-regulated kinases (ERK1/2) and increased intracellular Ca^2+^ levels were demonstrated to be part of the GPR55 signalling pathway [23]. Soon after, molecular docking studies identified several amino acids in the GPR55 sequence as anchoring sites for LPI [26, 45].

On this basis, we initially generated GPR55 alanine mutants of its most relevant LPI-binding residues: GPR55-K80A and GPR55-Q87A. Functional evaluation of these mutant proteins was carried out by transient transfection of HEK293T cells with constructs that coded for the HA-tagged human GPR55 proteins, as wild-type or mutated. When all of these showed similar cell-surface levels, as verified by FACS analysis (Additional file 3: Figure S1A), LPI stimulation induced comparable activation kinetics of ERK1/2 in all of the transfectants (Additional file 3: Figure S1B). Under these assay conditions, not only the *GPR55*-transfected, but also the empty-vector-transfected HEK293T cells showed LPI-stimulated increased phosphorylation of ERK1/2 (Additional file 3: Figure S1C).

We also evaluated different *GPR55*-transfection methods (lipid-mediated *versus* calcium phosphate) and agonists (soybean LPI *versus* synthetic purified LPI; see “Materials and methods” section for details). These showed inconsistent ERK1/2 activation in *GPR55*-transfected compared to empty-vector-transfected HEK293T cells, regardless of LPI concentration (100 nM to 10 µM) or length of stimulation (3–30 min).

In addition, HeLa cells were transfected with another *GPR55* construct, whereby this pcDNA3.1 vector included the optimised signal sequence (ss) for efficient cell-surface expression of the protein (here referred to as ssGPR55; see “Materials and methods” section for further details, and [40]). Also these cells showed similar activation upon LPI addition compared to empty-vector transfected cells for both ERK1/2 and AKT (Additional file 3: Figure S1D).

**Fig. 1:**
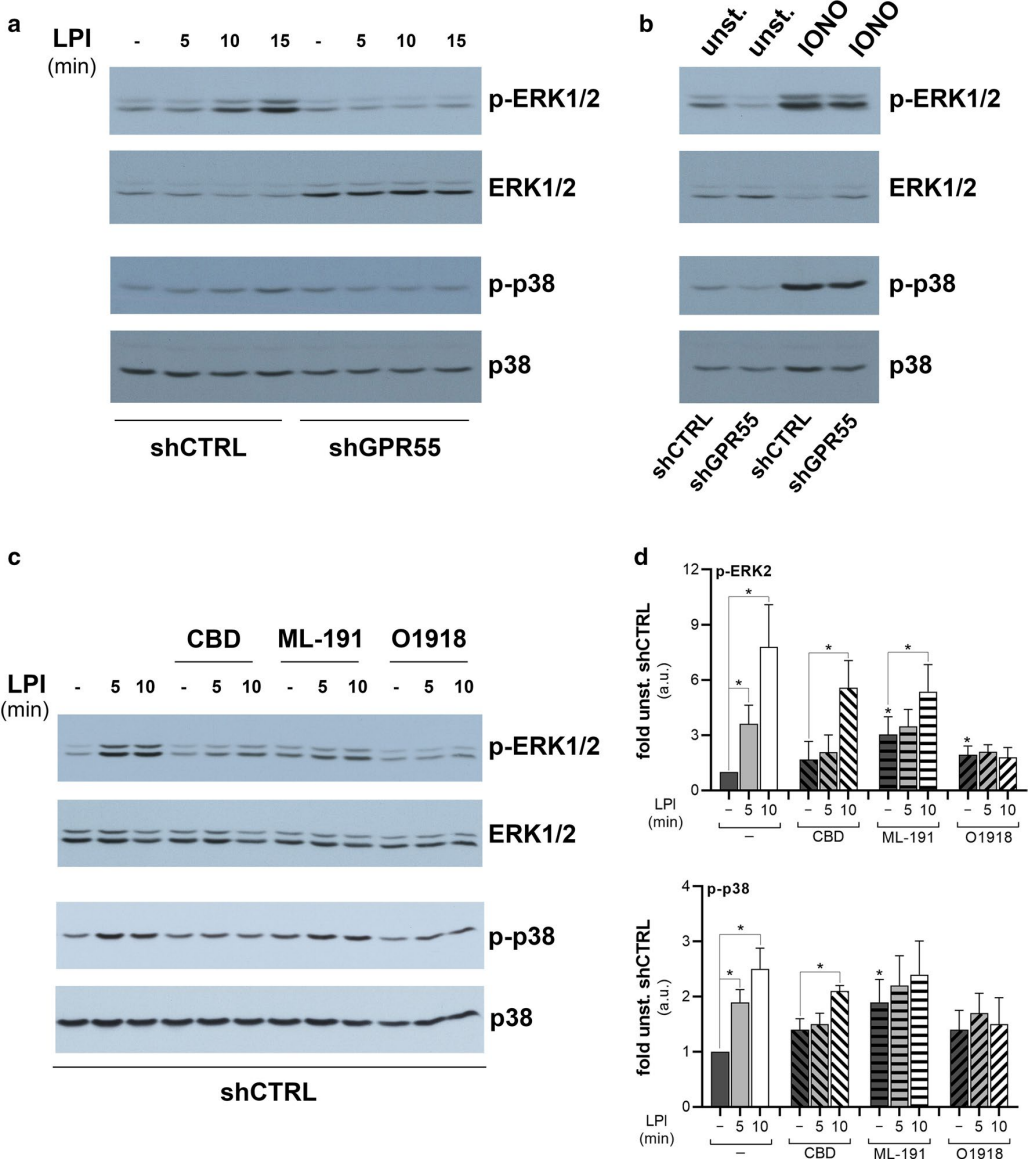
LPI activates ERK1/2 and p38 through an endogenous GPR55 in HeLa cells. HeLa cell clones obtained by control-interfering (shCTRL) or *GPR55*-interfering short-hairpin (shGPR55) transfection, were serum deprived for 2 h, then stimulated with 10 µM soybean LPI for the indicated times (**a**), or with 1 µM ionomycin (IONO) for 5 min (**b**). Western blotting of phosphorylated (p-ERK1/2, p-p38), and total ERK1/2 and p38 are shown, from an experiment representative of at least three independent. **c** shCTRL-HeLa were serum deprived for 2 h, then stimulated with 10 µM soybean LPI for the indicated times, in the absence or presence of 30 µM ML-191, 10 µM CBD or 10 µM O1918, for 10 min. Western blotting of phosphorylated (p-ERK1/2, p-p38), and total ERK1/2 and p38 are shown, from a representative experiment. **d** Densitometric analysis by arbitrary units (a.u.) of ERK2 (top) and p38 (bottom) phosphorylation levels, normalised for the correspondent protein levels. Data are expressed as fold unstimulated shCTRL-HeLa (unstim. shCTRL), and are means ± SE of three independent experiments. *p < 0.05 (Student’s *t*-tests) *versus* unstim. shCTRL (–) or corresponding unstimulated cells. unst., unstimunlated cells

Although LPI has been reported to trigger cell signalling in a GPR55-independent manner [46, 47], especially under non-stringent assay conditions, the present data were more suggestive of an endogenous GPR55 in HEK293T and HeLa cells that hindered the evaluation of the heterologous receptor mutants. To overcome this limitation, HeLa-cell clones that stably expressed a short hairpin (sh)RNA specific for the 5′-UTR of *GPR55* (shGPR55-HeLa clones) were produced, which showed 90% decreased expression of *GPR55* mRNA levels, as measured by quantitative real-time PCR. Any decrease in GPR55 protein levels could not be evaluated due to the lack of availability of any specific anti-GPR55 antibodies. In contrast to the control clones (shCTRL-HeLa clones), these shGPR55-HeLa clones did not show any ERK1/2 and p38 activation upon LPI addition, at least over the times evaluated here (Fig. 1a). However, they still activated these pathways on incubation with the Ca^2+^ ionophore ionomycin (Fig. 1b). Moreover, similar data were obtained by transient interference of* GPR55* using siRNAs in HeLa cells (data not shown), in support of the GPR55 specificity of these observed effects.

To determine whether LPI-induced signalling in shCTRL-HeLa clones was driven by endogenous GPR55 activation, the effects of three putative GPR55 antagonists were analysed: cannabidiol (CBD [22]), ML-191 [25] and O1918 [48] (Fig. 1c, d). All of these three compounds inhibited LPI stimulation of both ERK2 and p38 phosphorylation at 5 min, with significantly increased basal activation levels seen for ML-191 (for both ERK2 and p38) and O1918 (for ERK2). At 10 min of LPI stimulation in the control cells, ERK2 and p38 phosphorylation reached 7.8-fold and 2.5-fold basal levels, respectively. With addition of CBD, this LPI stimulation reached only 3.3-fold and 1.5-fold the CBD basal level, respectively, and for ML-191, only 1.8-fold and 1.2-fold the ML-191 basal level, respectively (Fig. 1c, d). Therefore, LPI-induced stimulation of ERK1/2 was strongly inhibited by CBD and ML-191, and abolished by O1918. The activation of ERK2 and p38 by ML-191 and O1918 in the absence of an agonist requires further investigation, but could be consequent to the modulation by these compounds of GPR55 interactions with other signalling molecules through the removal of inhibitory constraints, as shown for GPR55 dimerisation with CB_2_ [49, 50], although non-specific effects cannot be ruled out.

Overall, these data supported the hypothesis of functional endogenous GPR55 in the HEK2963T and HeLa cells, with reduced expression in the shGPR55-HeLa clones, which also no longer responded to the LPI treatment. In HeLa cells, although the LPI-triggered activation of ERKs and p38 was induced by a relatively high agonist concentration (10 µM), this was blunted by interference (Fig. 1a) and strongly inhibited by antagonism of GPR55 (Fig. 1c, d), suggesting that it mainly relies on activation of this receptor. On this assumption, similar conditions were used in the following assays.

### Lys^80^ is a requisite for LPI-stimulated GPR55 signalling

**Fig. 2:**
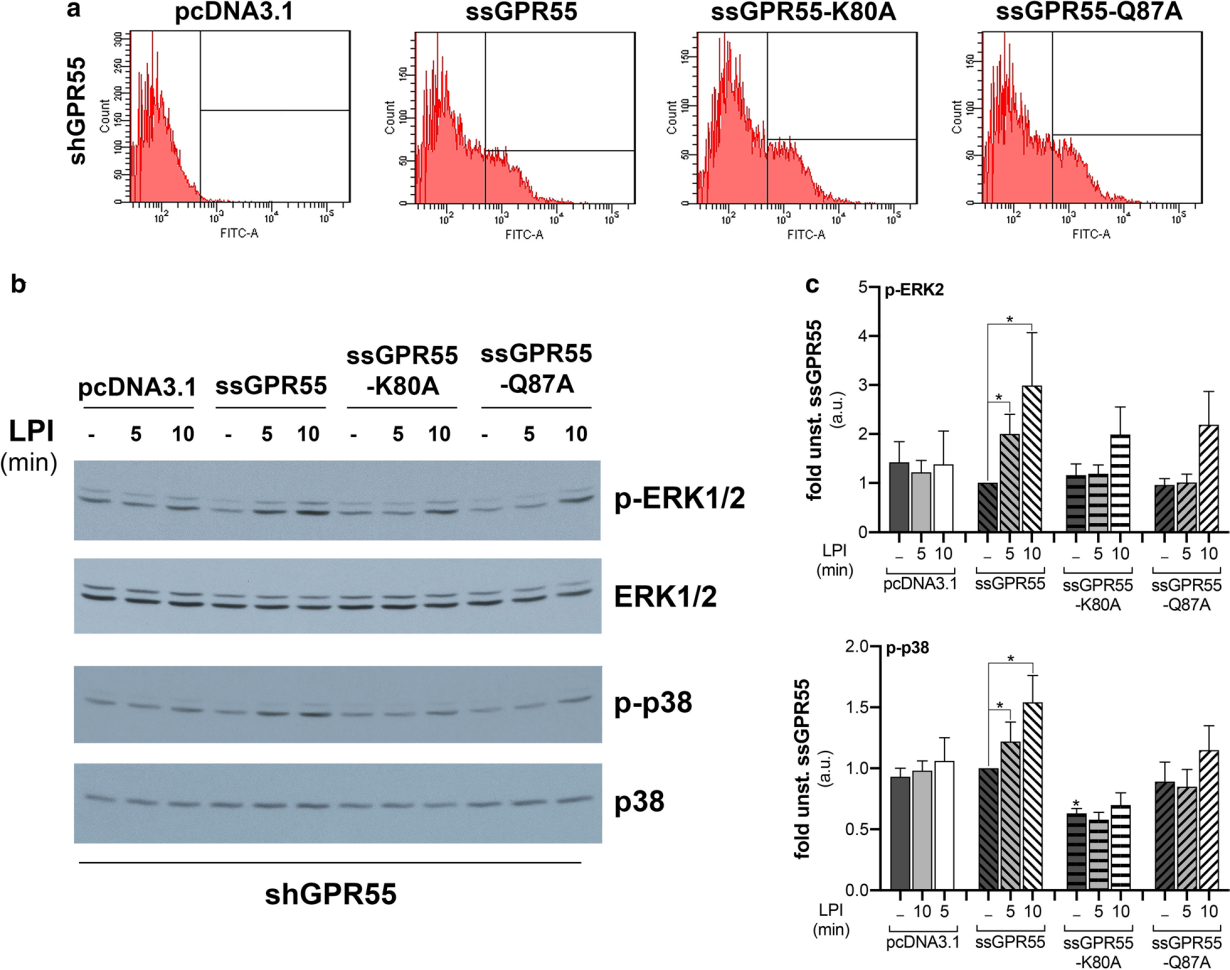
ssGPR55-K80A and ssGPR55-Q87A mutants have impaired LPI-induced stimulation of MAPKs. **a** FACS analysis with an anti-HA antibody of shGPR55-HeLa clones transfected with equal amounts (2.5 µg/well, in six-well-plates) of empty vector (pcDNA3.1), or of vector coding for ssGPR55 wild-type (ssGPR55) or the mutants (as indicated). The median fluorescence intensities and (% coefficient of variation) for these gated GPR55-transfected cells were: 1134 (132.5) for ssGPR55; 1286 (139.8) for ssGPR55-K80A; 1164 (134.8) for ssGPR55-Q87A. **b** Twenty-four hours after transfection, shGPR55-HeLa clones were serum deprived for 2 h, then stimulated with 10 µM 18:0 LPI for the indicated times. Western blotting of phosphorylated (p-ERK1/2, p-p38) and total ERK1/2 and p38 are shown from a representative experiment. **c** Densitometric analysis by arbitrary units (a.u.) of ERK2 (top) and p38 (bottom) phosphorylation levels, normalised for the correspondent protein levels. Data are expressed as fold of unstimulated ssGPR55-overexpressing cells (unst. ssGPR55), and are means ± SE of three independent experiments. *p < 0.05, (Student’s *t*-tests) *versus* corresponding unstimulated cells

The almost undetectable *GPR55* mRNA in the shGPR55-HeLa clones made these an ideal system to study the GPR55 mutant proteins. To this end, ssGPR55 (wild-type), ssGPR55-K80A and ssGPR55-Q87A were overexpressed in the shGPR55-HeLa clones, where they reached comparable levels at the plasma membrane (Fig. 2a). Differently from the ssGPR55-expressing shGPR55-HeLa clones, the clones transfected with the empty vector (pcDNA3.1) did not respond to LPI addition over the time analysed, while for expression of both the ssGPR55-K80A and ssGPR55-Q87A mutants there was impaired LPI-stimulated activation of ERK2 (no response at 5 min, reduced response at 10 min), and no LPI-stimulated activation of p38 (Fig. 2b, c).

**Fig. 3:**
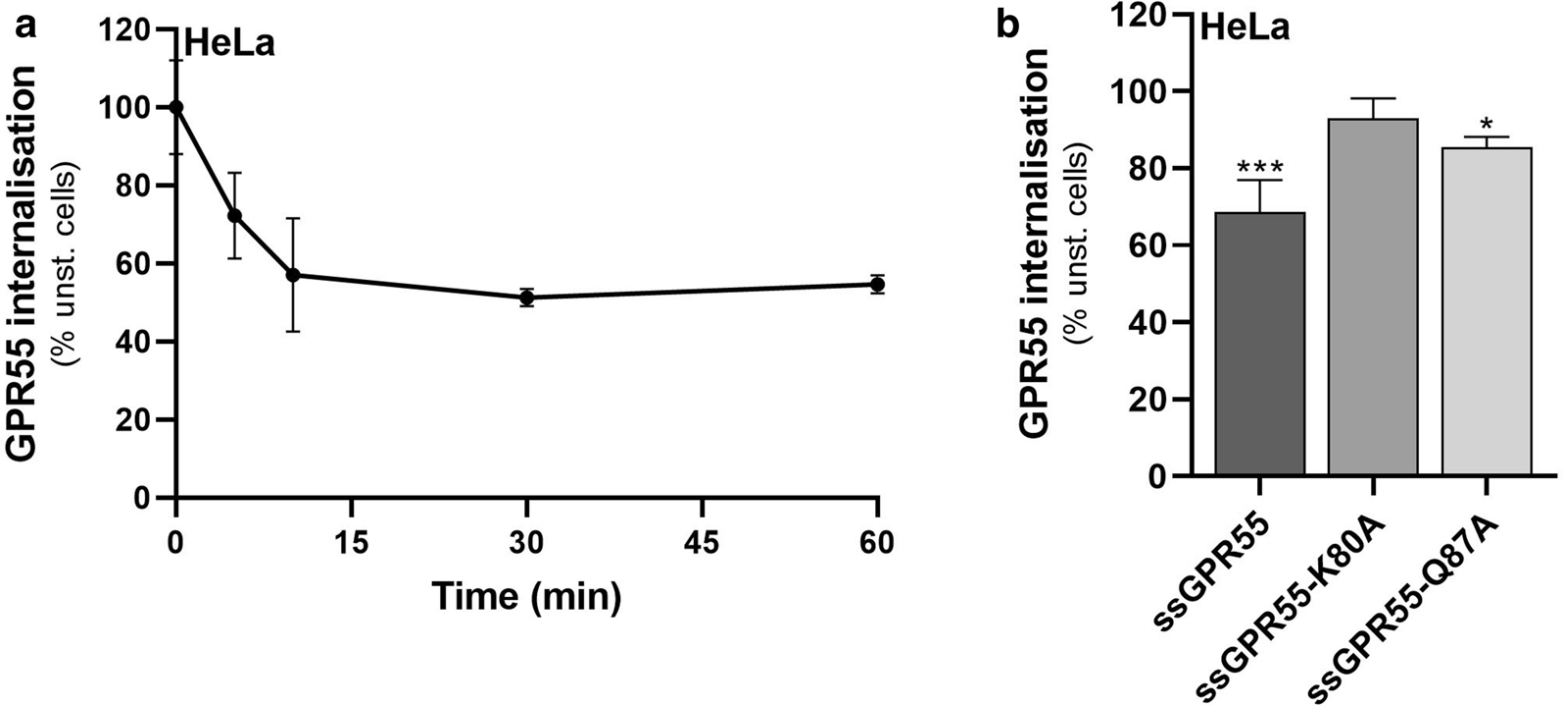
LPI does not induce GPR55-K80A internalisation. **a** Quantification of ssGPR55 internalisation in transfected HeLa cells by FACS analysis, after live-cell immunostaining using the murine anti-HA antibody (see “Materials and methods” section for details). Cells were stimulated or not with 10 µM soybean LPI and analysed over time. The mean fluorescences obtained are expressed as percentages of the unstimulated sample (unst.), and are indicative of the residual GPR55 plasma-membrane localisation. The efficiency of transfection in these experiments was 55%, and the mean fluorescence of ssGPR55-expressing unstimulated HeLa cells was 3305 ± 397 a.u.. Data are means ± SEM of three independent experiments. **b** HeLa cells expressing ssGPR55 wild-type and mutants (as indicated) were stimulated for 15 min without or with 10 µM soybean LPI, and then cell-surface localisation of the receptors was quantified by FACS. Data are means ± SEM of three independent experiments, and are mean fluorescences of each sample as percentage of correspondent unstimulated cells. The efficiency of transfection in these experiments was 44%, 42%, 42%, and the mean fluorescences were 1982 ± 247, 1747 ± 325, 1889 ± 443, for ssGPR55 wild-type, ssGPR55-K80A and ssGPR55-Q87A, respectively. *p < 0.05; ***p < 0.005 (Student’s *t*-tests) *versus* corresponding unstimulated cells

Prolonged LPI stimulation induces GPR55 down-regulation from the plasma membrane [38]. Time-course of LPI-induced internalisation of overexpressed ssGPR55 in HeLa cells indicated that this process started in the first few minutes of LPI stimulation, and reached a plateau after 10 min (Fig. 3a). At comparable expression levels as the wild-type ssGPR55 receptor, both the ssGPR55-K80A and ssGPR55-Q87A mutants showed reduced internalisation. Indeed, 15-min stimulation with LPI reduced the cell-surface levels of the HA-tagged wild-type ssGPR55 receptor by 30%, while the cell-surface levels of ssGPR55-Q87A were reduced by only 15%, and those of ssGPR55-K80A were not changed (Fig. 3b).

Both these analyses of LPI-induced MAPK activation and GPR55 internalisation indicated that the ssGPR55-K80A and ssGPR55-Q87A mutants were less responsive to this agonist; indeed, the ssGPR55-K80A mutation greatly impaired LPI activity, which is in support of a role for Lys^80^ for LPI binding to the human GPR55 receptor sequence.

### GPR55 mediates LPI-induced Ca^2+^ increases and reorganisation of the actin cytoskeleton in RAW264.7 cells

One of the main phenotypes of the *Gpr55*-knockout mice was an increase in trabecular bone mass compared to the wild-type mice, which was suggestive of impaired osteoclast functions [9]. To determine the role of GPR55 in osteoclastogenesis, we took advantage of a well-validated in-vitro model of osteoclast differentiation that is based on RAW264.7 monocytes/macrophages as osteoclast precursor cells [51, 52]. To assess the suitability of this model, we verified that GPR55 was functionally active in RAW264.7 cells, by monitoring the LPI-induced and GPR55-dependent effects on intracellular Ca^2+^ levels and actin cytoskeleton reorganisation.

**Fig. 4:**
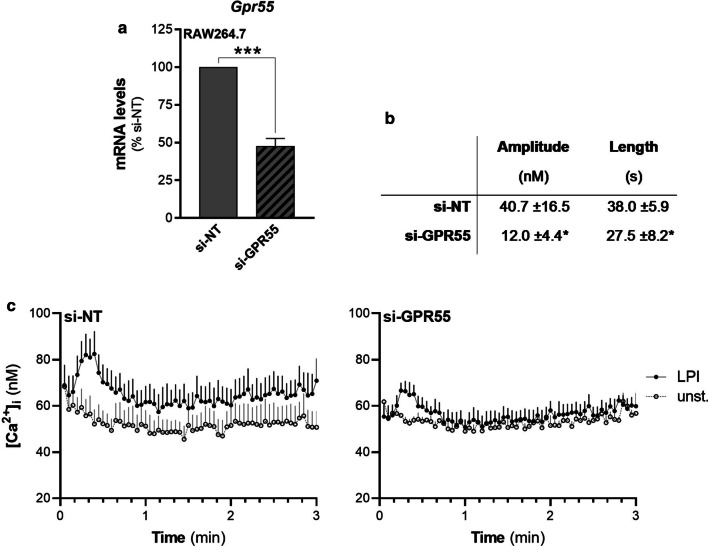
LPI induces increases in intracellular Ca^2+^ through an endogenous GPR55 in RAW264.7 cells. **a*** Gpr55* mRNA levels of RAW264.7 cells interfered with non-targeting (si-NT) or* Gpr55*-specific (si-GPR55) siRNAs were quantified by real-time PCR and normalised using *β*_*2*_*-microglobulin* expression, as the housekeeping gene. Data are means ± SEM from seven independent experiments. **b**, **c** Interfered RAW264.7 cells were serum deprived for 1 h, loaded with Fluo4-NW, and then stimulated without or with 10 µM 16:0 LPI. Changes in fluorescence of each well of a 96-well plate were recorded, and the intracellular Ca^2+^ concentrations were calculated (see “Materials and methods” section). **b** Comparison between si-NT-and si-GPR55-responses to 10 µM 16:0 LPI, as means of six independent experiments performed in quadruplicates. Amplitude (height of the Ca^2+^ peak measured at the apex) and duration (length of the peak measured at the bases) of the responses were measured. **c** Data are means ± SE of quadruplicates within a representative experiment. *p < 0.05; ***p < 0.005 versus si-NT (Student’s *t*-tests)

To this end, RAW264.7 cells were treated with non-targeting or *Gpr55*-specific siRNAs (si-NT, si-GPR55, respectively), where this GPR55 silencing resulted in about 50% reduction in *Gpr55* mRNA levels, according to quantitative real-time PCRs (Fig. 4a). However, the correspondent decrease in GPR55 protein levels could not be evaluated due to the lack of any specific anti-GPR55 antibodies.

Comparative analysis of these cells allowed evaluation of LPI-stimulated GPR55-dependent processes. In Ca^2+^ assays, within the first minute of LPI addition to the RAW264.7 cells after 72 h of interference with si-NT, a peak in intracellular Ca^2+^ levels was observed, with a mean increase of ~ 40 nM over basal levels (Fig. 4b, c). In si-GPR55–treated cells, this Ca^2+^ increase was significantly reduced in both amplitude and duration (Fig. 4b, c).

**Fig. 5:**
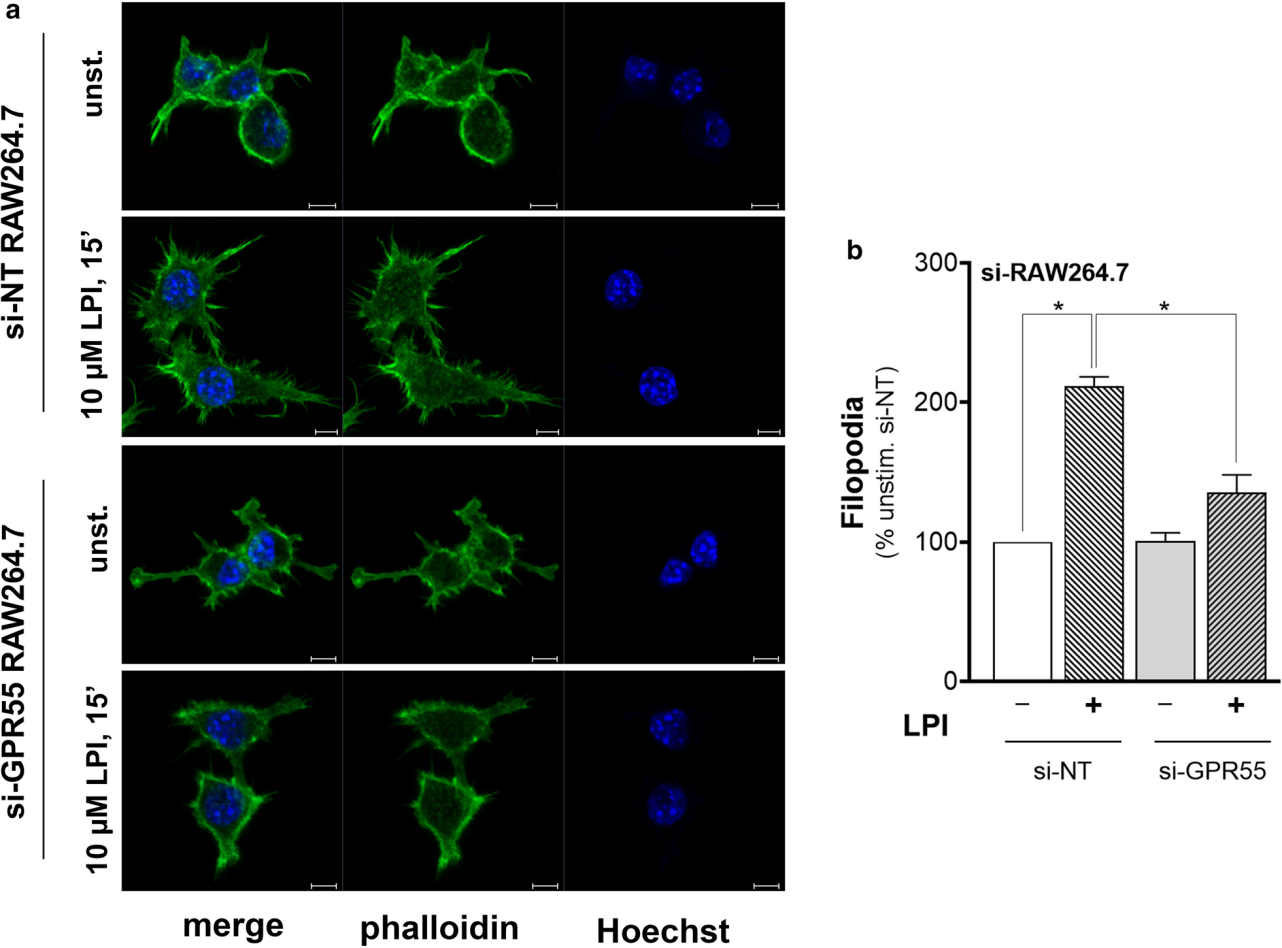
LPI induces filopodium appearance through endogenous GPR55 in RAW264.7 cells. RAW264.7 cells interfered with non-targeting (si-NT) or *Gpr55*-specific (si-GPR55) siRNAs were serum deprived for 2 h, stimulated without or with 10 µM soybean LPI for 15 min, fixed and stained for confocal imaging. Phalloidin staining allows visualisation of filamentous actin, while Hoechst staining reveals cell nuclei (see “Materials and methods” section for details). **a** Representative confocal images of actin staining, as indicated. Scale bars, 5 µm. **b** Quantification of filopodium formation, as described in “Materials and methods” section. Data are expressed as proportions (%) of the unstimulated si-NT, as means (± SEM) of three independent experiments, each carried out in duplicate. *p < 0.05 *versus* LPI-stimulated si-NT (one-way ANOVA, followed by Tukey test)

To monitor the effects on the actin cytoskeleton, these same cell systems were analysed by confocal microscopy, after fixation and staining with fluorescent phalloidin. In the unstimulated cells, those treated with si-GPR55 showed different morphology compared to the control unstimulated si-NT–treated cells, with increased fluorescence at the cell periphery, resembling actin ruffling (Fig. 5a). Then, LPI stimulation induced a time-dependent increase in the numbers of filopodia in the si-NT–interfered RAW264.7 cells (Fig. 5b). Instead, in the si-GPR55–treated cells, there were no significant signs of actin cytoskeleton reorganisation after LPI addition (Fig. 5b).

**Fig. 6:**
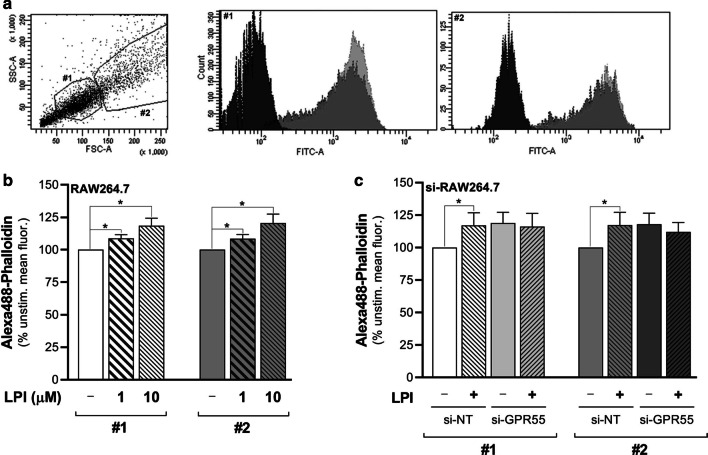
LPI induces actin cytoskeleton remodelling through endogenous GPR55 in RAW264.7 cells. **a** FACS analysis dot plot of RAW264.7 cells (left) shows two main cell sub-populations (#1, #2) with different intrinsic features in both cell size and granularity, which were separately gated. Fluorescence distributions of #1 and #2 (right). Black, unstained cells; dark/light grey, unstimulated/LPI-stimulated phalloidin-stained RAW264.7 cells. **b** Phalloidin-stained wild-type RAW264.7 cells stimulated without or with 1 µM or 10 µM soybean LPI. Changes in mean fluorescences of the #1 and #2 cell sub-populations were recorded by FACS analysis. Data are mean fluorescences as percentages of unstimulated cell fluorescence, as means ± SE of at least five independent experiments. **c** Phalloidin-stained si-NT-interfered or si-GPR55-interfered RAW264.7 cells stimulated without and with 10 µM soybean LPI. Changes in mean fluorescences of the #1 and #2 cell sub-populations were recorded by FACS analysis. Data are mean fluorescences as percentages of unstimulated-si-NT-cell fluorescence, as means ± SE of five independent experiments. *p < 0.05 (Student’s *t*-tests)

For quantitative analysis of the LPI effects on filamentous actin, the RAW264.7 cells were analysed by FACS. This analysis defined two main sub-populations of the RAW264.7 cells (Fig. 6a, #1, #2) that showed different intrinsic features in both cell size and granularity. Upon LPI addition, these two sub-populations showed similarly increased Alexa488-phalloidin mean fluorescence, which depended on LPI concentration and time of stimulation, with a maximal 20% increase seen with 10 µM LPI stimulation for 15 min (Fig. 6a, b). To determine whether this effect was dependent on GPR55 activation, the si-NT–treated and si-GPR55–treated cells were compared. In the si-NT cells, LPI addition resulted in a significant increase in the mean phalloidin fluorescence for both of the cell sub-populations (Fig. 6c), which was comparable to the wild-type RAW264.7 macrophages (Fig. 6b). Conversely, the si-GPR55 cells showed higher basal mean phalloidin fluorescence that did not increase further on LPI addition (Fig. 6c).

These data thus indicated that LPI activated GPR55 in RAW264.7 cells, which resulted in increased intracellular Ca^2+^ levels and in actin cytoskeleton remodelling, with the appearance of filopodia and a general increase in filamentous actin.

### GPR55 contributes to the osteoclastogenesis of precursor RAW264.7 cells

The expression of a functionally active GPR55 in RAW264.7 cells, which are osteoclast precursors, support their use to investigate GPR55 role in the osteoclastogenesis process.

**Fig. 7:**
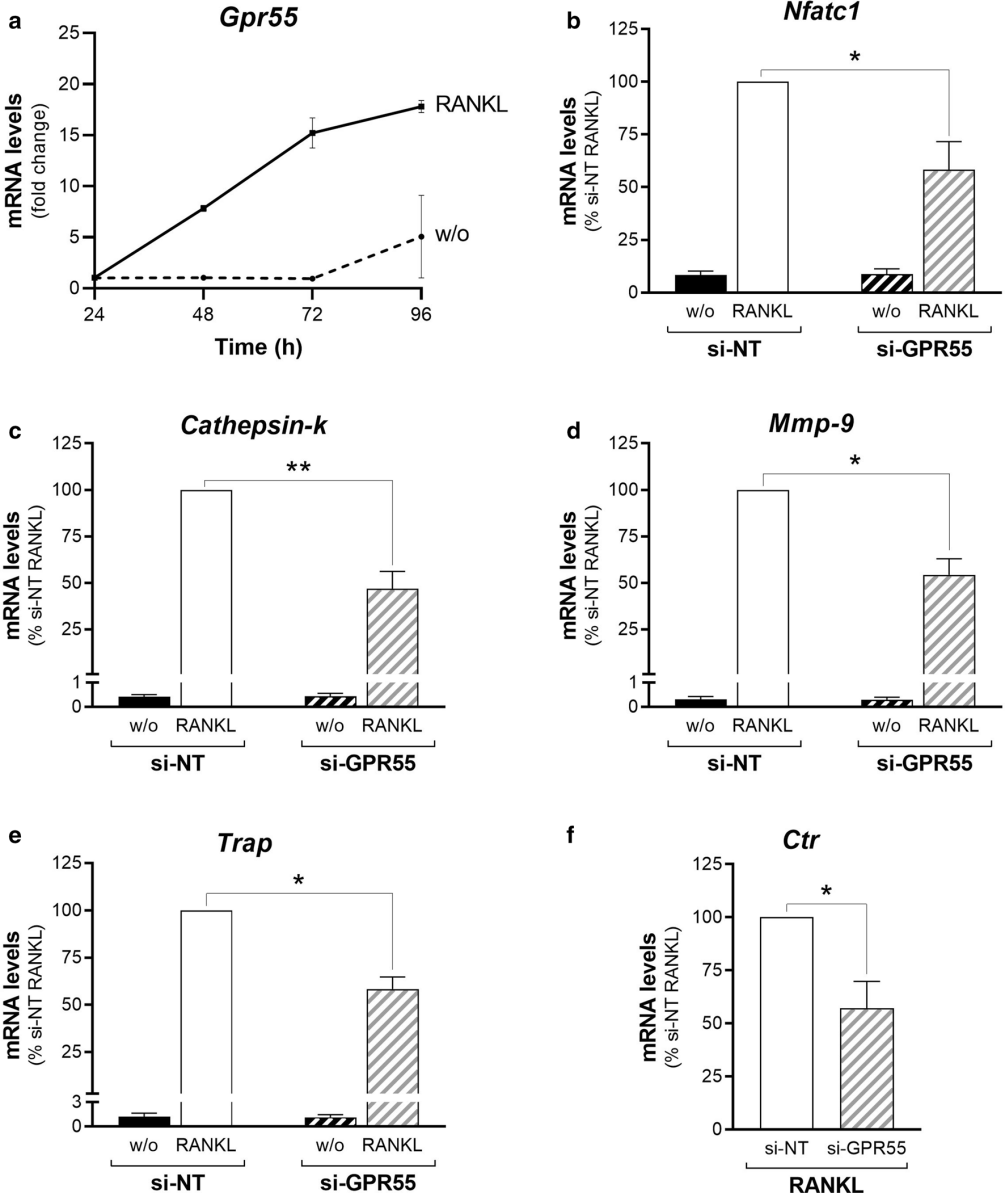
Reciprocal regulation of GPR55 expression and RANKL-induced osteoclastogenesis of RAW264.7 macrophages. **a** Time-course of *Gpr55* mRNA levels in precursor RAW264.7 cells in absence (w/o) or presence of 30 ng/mL RANKL. Transcripts were quantified by real-time PCR and normalised using *β*_*2*_*-microglobulin* expression, as the housekeeping gene. Data are means ± SEM from three independent experiments. **b**–**f** Real-time PCR analysis of osteoclastogenesis markers (as indicated) in RAW264.7 cells interfered with non-targeting (si-NT) or *Gpr55*-targeting (si-GPR55) siRNAs, and subsequently treated without (w/o) or with 15–30 ng/mL RANKL for 72 h. The transcripts were quantified and normalised using *β*_*2*_*-microglobulin* expression, as the housekeeping gene. Data are means ± SEM from four independent experiments. *p < 0.05; **p < 0.01 (Student’s *t*-tests). w/o, cells incubated without RANKL; RANKL, RANKL-differentiated cells

Three-day treatments of RAW264.7 cells with the cytokine ‘receptor-activator of nuclear factor kappa-β ligand’ (RANKL) led to the formation of multinucleated, functionally active, osteoclasts (see “Materials and methods” section for details; [52]). For the RAW264.7 cells in the absence of RANKL, *Gpr55* mRNA levels remained unchanged during the initial 72 h, and then had increased by 96 h (Fig. 7a), when signs of spontaneous osteoclast differentiation started to appear. Instead, during the RANKL-promoted osteoclastogenesis, *Gpr55* transcription was substantially enhanced in a time-dependent manner, with a 14-fold increase in its mRNA levels at 72 h, which then remained stable over the following 48 h of RANKL treatment. Although information on the GPR55 protein levels is lacking here due to the low sensitivity and specificity of the commercially available antibodies, the observed modulation of the GPR55 receptor at the mRNA level during the RANKL treatment was suggestive of GPR55 involvement in osteoclastogenesis of RAW264.7 cells.

To investigate this hypothesis further, the effects on osteoclast differentiation of *Gpr55* silencing were evaluated. The osteoclast precursors, the RAW264.7 cells, were interfered with the si-NT or si-GPR55 siRNAs, where the targeting siRNAs reduced the *Gpr55* mRNA levels by 50%, when monitored by quantitative real-time PCR (Additional file 2: Table S2). As controls, the cannabinoid (*Cb1, Cb2*) and lysophosphatidic acid (*Lpar1*) receptors were also analysed, due to their homology with GPR55 and their involvement in the osteoclastogenesis process [53, 54]. However, no significant modulation of the mRNA levels of the cannabinoid or lysophosphatidic acid receptors was seen by GPR55 silencing (Additional file 1: Table S2). These interfered cells were then incubated without or with RANKL, to obtain fully differentiated osteoclasts. The osteoclast maturation was monitored during the entire RANKL treatment by quantitative real-time PCR, to quantify the mRNA expression levels of five representative differentiation markers: ‘nuclear factor of activated T-cells, cytoplasmic 1′ (NFATc1), as an early osteoclastogenesis marker; matrix metalloproteinase-9 (MMP-9) and cathepsin-K protease as intermediate; and tartrate-resistant acid phosphatase (TRAP) and calcitonin receptor (CTR) as two late osteoclastogenesis markers (Additional file 4: Figure S2). The transient silencing of *Gpr55* significantly impaired RANKL-induced transcription of all of these five differentiation markers, with their mRNA levels at 72 h showing 40% reduction for *Nfatc1*, *Trap* and *Ctr*, 45% reduction for *Mmp-9*, and 55% reduction for *Cathepsin-k*, without any effects on their basal transcription (Fig. 7b–f).

**Fig. 8:**
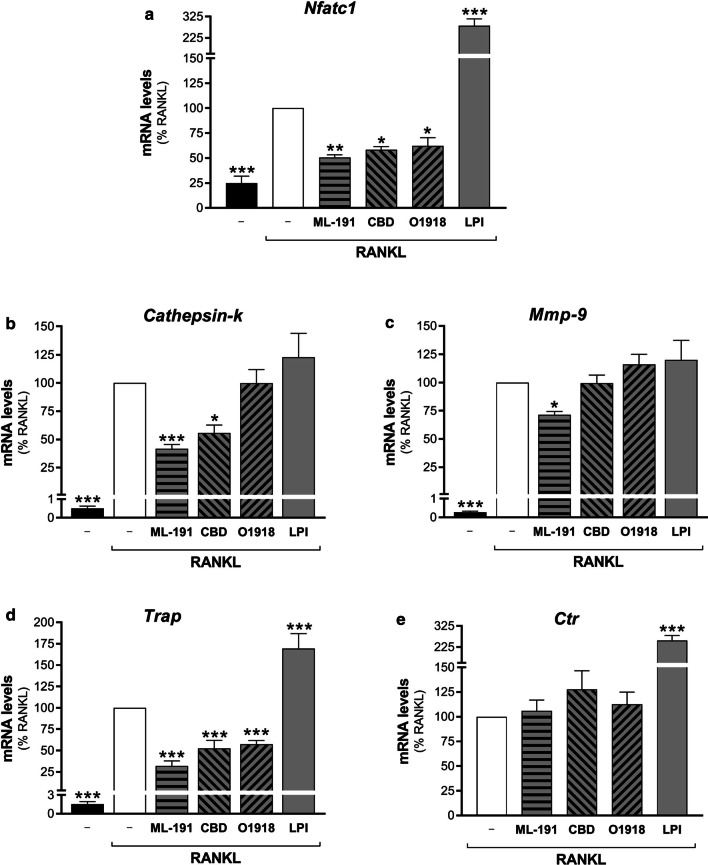
Effects of GPR55 modulators on osteoclast maturation. Real-time PCR analysis of the differentiation markers (as indicated) in RAW264.7 cells treated in the absence (–) or presence of 15–30 ng/mL RANKL for 72 h, without or with GPR55 putative antagonists/agonist (0.5 µM ML-191, 0.5 µM CBD, 30 µM O1918, 1 µM soybean LPI). Transcripts were quantified and normalised using *β*_*2*_*-microglobulin* expression, as the housekeeping gene. Data are means ± SEM of four independent experiments. *p < 0.05; **p < 0.01, ***p < 0.005 versus RANKL (one-way ANOVA, followed by Fisher’s least significant difference tests)

In addition to the molecular approach, a role for GPR55 in osteclastogenesis was strengthened using complementary pharmacological tools. During differentiation of the RAW264.7 cells with RANKL, they were also treated with the regulators of GPR55 signal transduction: ML-191, CBD, O1918, as putative GPR55 antagonists; and LPI as an agonist. The effects of these treatments on both osteoclast maturation and fusion were determined. A substantial block of RANKL-induced transcription of almost all of the differentiation markers was induced by ML-191 at 72 h, with 50% reduction in the mRNA levels for *Nfatc1*, 60% for *Cathepsin-k*, 30% for *Mmp-9* and 70% for *Trap*, while *Ctr* mRNA levels were not affected (Fig. 8). CBD blocked RANKL-induced transcription at 72 h, with 40% reduction in the mRNA levels for *Nfatc1*, 44% for *Cathepsin-k*, and 50% for *Trap*, while *Mmp-9* and *Ctr* mRNA levels were not affected (Fig. 8). O1918 blocked RANKL-induced transcription with 40% reduction in the mRNA levels for *Trap* and *Nfatc1*, while *Cathepsin-k*, *Mmp-9* and *Ctr* mRNA levels were not affected (Fig. 8).

The GPR55 agonist LPI further stimulated RANKL-induced transcription of *Nfatc1* by 0.2-fold (24 h), 1.1-fold (48 h), and 1.8-fold (72 h; Fig. 8a), of *Trap* by 0.2-fold (24 h), 0.7-fold (48 h), and 0.7-fold (72 h, Fig. 8d), and of *Ctr* by 2.4-fold (48 h) and 1.6-fold (72 h; Fig. 8e). For *Cathepsin-k* transcription, no modulation was seen for LPI at 72 h of RANKL treatment (Fig. 8b); however, further analysis of the transcription kinetics during RAW264.7 osteoclastogenesis showed that *Cathepsin-k* levels reached a plateau at this differentiation stage (Additional file 4: Figure S2). Instead, LPI addition stimulated RANKL-induced transcription of *Cathepsin-k* at the earlier times of 24 h and 48 h, with increases in the mRNA levels of 1.4-fold and 1.2-fold, respectively. Moreover, LPI had no effects on *Mmp-9* transcription at any of the times analysed (Fig. 8c, 72 h). As for the other treatments, LPI was present during the entire differentiation, as its washout after the initial 24 h did not produce any significant effects on osteoclastogenesis marker transcription (data not shown).

To verify that the above effects of LPI depended on GPR55, two specific GPR55 antagonists, ML-191 and CID16020046 [55], were added, and the RANKL-induced transcription of two representative markers, *Nfatc1* and *Ctr*, was monitored. Whereas LPI-stimulated transcription of *Nfatc1* was completely blocked by the two antagonists, that of *Ctr* was blocked by ML-191 and only 50% reduced by CID16020046 (Additional file 5: Figure S3A, B). Therefore, LPI modulation of *Ctr* transcription was verified also in RANKL-differentiated cells silenced for GPR55. As a further control, a different GPR55 agonist, ML-184 [25], was used in these assays. Both LPI and ML-184 similarly increased RANKL-induced transcription of *Ctr* only in precursor cells treated with non-targeting siRNAs, and not in those silenced for GPR55 (Additional file 5: Figure S3C).

The pharmacological treatments also modulated GPR55 expression levels. The RANKL-stimulated mRNA levels of *Gpr55* at 72 h were further enhanced by 1.8-fold in presence of the most specific antagonist ML-191, by 0.7-fold with the less selective antagonist CBD, while O1918 did not show any effects (Table 1). Also, LPI increased *Gpr55* expression levels by 0.5-fold (Table 1).

**Table 1:** Modulation of *Gpr55* mRNA levels by receptor ligands

Condition	Modulator	*Gpr55* mRNA levels (fold non-differentiated)
w/o	–	1.0***
+ RANKL	–	16.1 ± 1.6
	+ 1 μM soybean LPI	24.5 ± 2.1**
	+ 0.5 μM ML-191	46.1 ± 5.1***
	+ 0.5 μM CBD	27.7 ± 0.5***
	+ 30 μM O1918	15.8 ± 3.1

RAW264.7 cells were treated without (w/o) or with 30 ng/mL RANKL for 72 h, in the absence or presence of the indicated GPR55 modulators

Data are means ± SEM of at least three independent experiments. **p < 0.01; ***p < 0.005 versus RANKL alone (one-way ANOVA, followed by Fisher’s least significant difference tests)

*CBD* cannabidiol

**Fig. 9:**
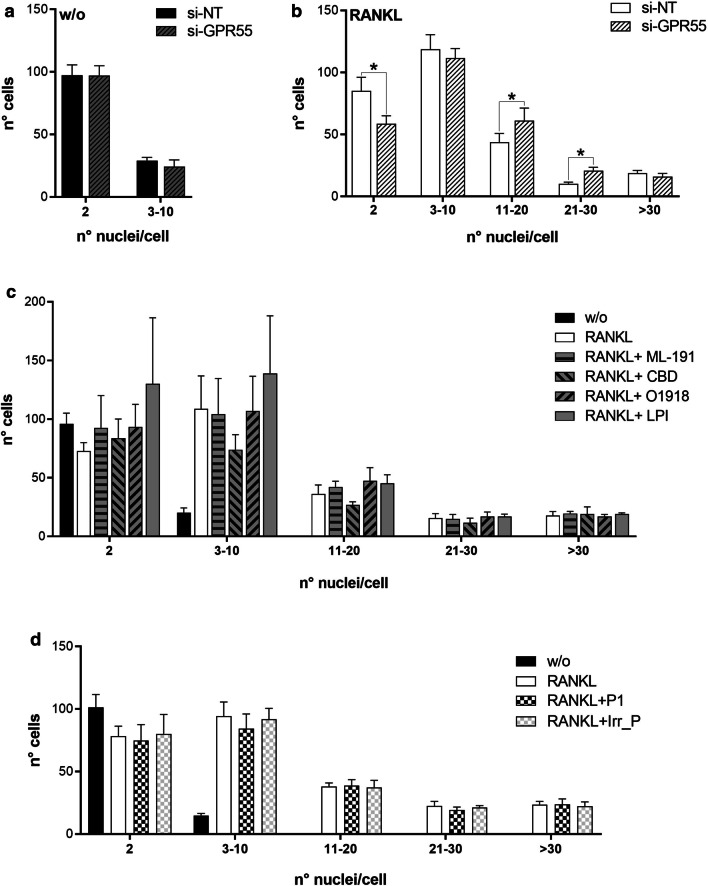
Effects of GPR55 interference and GPR55 modulators on osteoclast syncytia. RAW264.7 cells were interfered using non-targeting (si-NT) or *Gpr55*-targeting (si-GPR55) siRNAs, and then treated without (w/o) (**a**) or with 15–30 ng/mL RANKL (**b**). Osteoclast syncytia formation was determined after 72 h as number of nuclei/cell, under fluorescence microscopy. **c**, **d** RAW264.7 cells were treated without (w/o) or with 15–30 ng/mL RANKL, in the absence or presence of the GPR55 putative antagonists/agonist (0.5 µM ML-191, 0.5 µM CBD, 30 µM O1918, 1 µM soybean LPI) (**c**), or of 150 nM (0.2 µg/mL) peptides (Peptide-P1, P1; the irrelevant peptide with the sequence CGGNGPGLC, Irr_P) (**d**). Osteoclast syncytium formation was determined after 72 h of RANKL treatment as the number of nuclei/cell, under fluorescence microscopy. Data are means ± SE of three independent experiments. *p < 0.05 (Student’s *t*-tests). w/o, cells incubated without RANKL; RANKL, RANKL-differentiated cells

In parallel assays, the efficiency of osteoclast-syncytium formation by the interfered osteoclast precursor cells (si-NT and si-GPR55 RAW264.7 cells) was evaluated, by quantification of the number of nuclei per cell using fluorescence microscopy (see “Materials and methods” section for details). *Gpr55* silencing with si-GPR55 did not affect the numbers of nuclei per cell of the undifferentiated RAW264.7 cells (Fig. 9a). However, with RANKL treatment, *Gpr55* silencing promoted significant increases in multinucleated osteoclasts with 11–30 nuclei, with a concomitant significant reduction on the proportion of bi-nucleated cells (Fig. 9b). No significant effects were seen with the addition of the GPR55 antagonists and agonist, compared to RANKL alone (Fig. 9c, Additional file 6: Figure S4).

These data thus indicated the involvement of GPR55 in transcriptional remodelling driven by RANKL in these precursor RAW264.7 cells, where GPR55 expression was essential for efficient osteoclast maturation, as demonstrated by GPR55 silencing. The GPR55 signal transduction pathway modulated the marker transcription in different ways, with the GPR55 antagonist behaviours ascribable to their own specificity and selectivity. However, for osteoclast fusion, which is a late differentiation event, this was not modulated by GPR55 signalling. Instead, decreased *Gpr55* mRNA levels were associated with larger (more multinucleated) osteoclasts, in line with what was reported for osteoclasts that were derived from precursor cells of *Gpr55*-knockout mice [9].

### GPR55-specific peptides regulate osteoclast maturation of precursor RAW264 cells

In a previous study, we succeeded in targeting GPR55 with peptides using whole-cell-based screening of a phage-displayed random library. The bait used was HEK293 cells that heterologously expressed human GPR55, with a library of cyclic peptides of seven residues that contained two flanking cysteines presented by M13 phages [38]. Among these peptides seen to bind to GPR55, peptide-P1 (CKKNSPTLC) inhibited GPR55-dependent proliferation of two human B-lymphoblastoid cell lines [38]. To determine whether peptide-P1 can regulate RAW264.7 osteoclastogenesis, validation of its recognition of murine GPR55 was initially required. Although peptide-P1 shows specificity for the human receptor [38], human and murine GPR55 share protein sequence identity of only 75% (84% similarity; NP_005674.2 vs NP_001028462.2). For this validation, binding of fluorescein-isothiocyanate (FITC)-conjugated peptide-P1 (FITC-P1) to intact RAW264.7 cells was monitored over time by FACS (Additional file 7: Figure S5A). From the FITC-P1 binding curve, an affinity binding constant of 22.7 µM was extrapolated, which was close to its affinity towards human GPR55 (20 µM, [38]).

The specificity of peptide-P1 binding towards murine GPR55 was further analysed in RAW264.7 cells interfered or not for GPR55. A co-transfection (with the siRNAs) of the fluorescent siGLO-Red transfection indicator helped to increase the sensitivity of this analysis, by following the interfered cells (see “Materials and methods” section for details). FITC-P1 showed a 26.5% decrease in binding to RAW264.7 cells silenced for *Gpr55* (si-GPR55 + siGLO) relative to control cells (si-NT + siGLO), with the binding of the scrambled peptide not significantly affected by *Gpr55* interference (Additional file 7: Figure S5B).

**Fig. 10:**
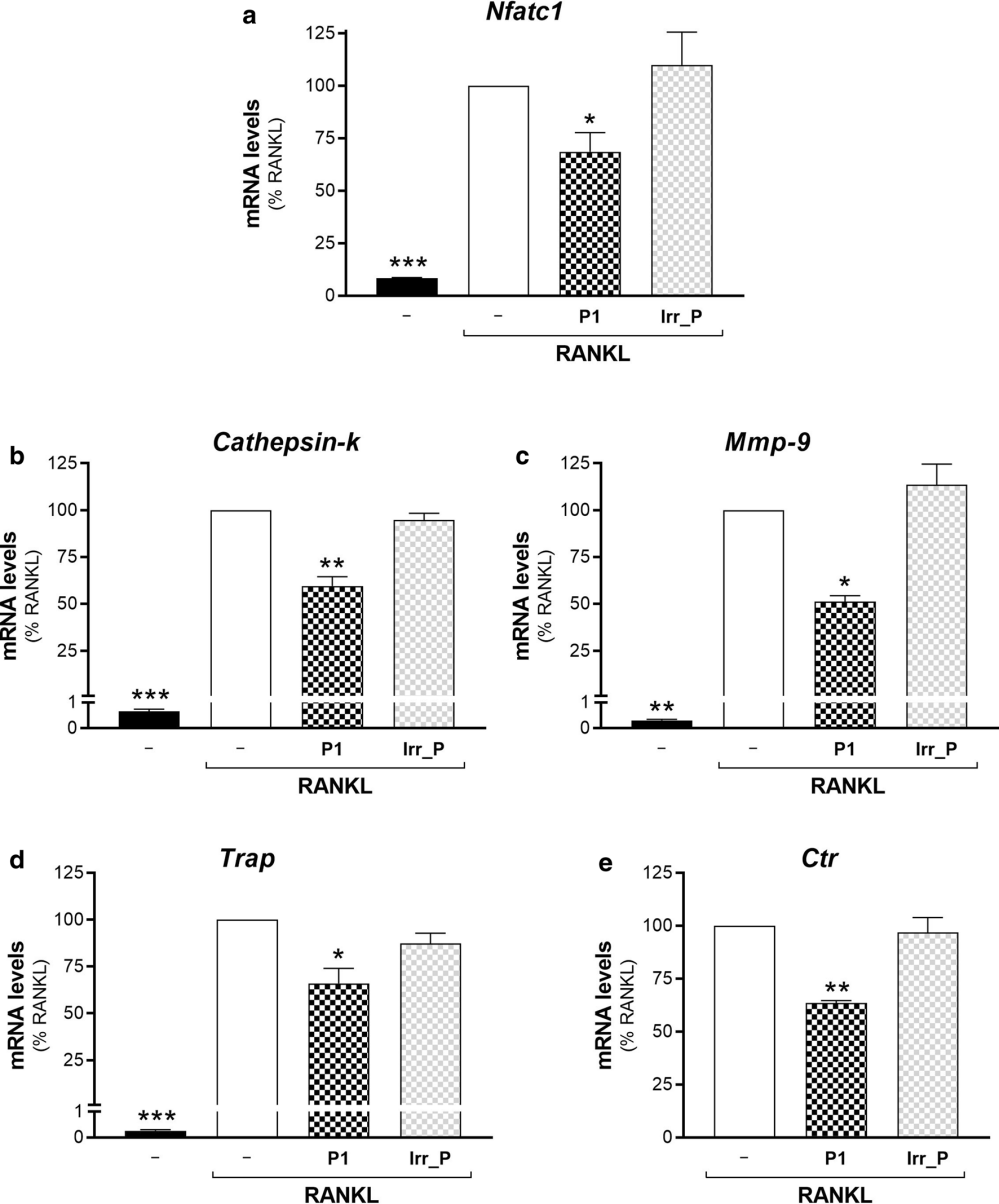
Peptide-P1 inhibits osteoclast maturation. Real-time PCR analysis of the differentiation markers (as indicated) in RAW264.7 cells treated without (–) or with 15 ng/mL RANKL for 72 h, in absence or in presence of 150 nM (0.2 µg/mL) Peptide-P1 (P1), or an irrelevant peptide (Irr_P, CGGNGPGLC). Transcripts were quantified and normalised using *β*_*2*_*-microglobulin* expression, as the housekeeping gene. Data are means ± SEM of three independent experiments. *p < 0.05; **p < 0.01; ***p < 0.005 versus RANKL (one-way ANOVA, followed by Fisher’s least significant difference tests)

To evaluate the effects of peptide-P1 on osteoclast maturation and fusion, during the entire differentiation of the RAW264.7 cells with RANKL, they were treated without any peptide or with 150 nM (0.2 µg/mL) peptide-P1 or an irrelevant control peptide (Irr_P, CGGNGPGLC). As for the other modulators of GPR55, the treatment with peptide-P1 did not have any significant effects on osteoclast syncytium formation induced by RANKL (Fig. 9d). Instead, as shown by the mRNA levels of the five differentiation markers, and unlike the Irr_P treatment, the treatment with peptide-P1 induced significant inhibition of osteoclast maturation: 30% reduction for *Nfatc1*, 40% for *Cathepsin-k*, 50% for *Mmp-9* and 35% for *Trap* and *Ctr* (Fig. 10).

These data showed an inhibitory effect of peptide-P1 in osteoclast maturation, a process previously shown to be dependent on GPR55 signalling, and not in the fusion step during osteoclast syncytium formation, which was not affected even by GPR55 agonists/antagonists.

### GPR55 role in bone resorption of osteoclasts derived from RAW264.7 cells

**Fig. 11:**
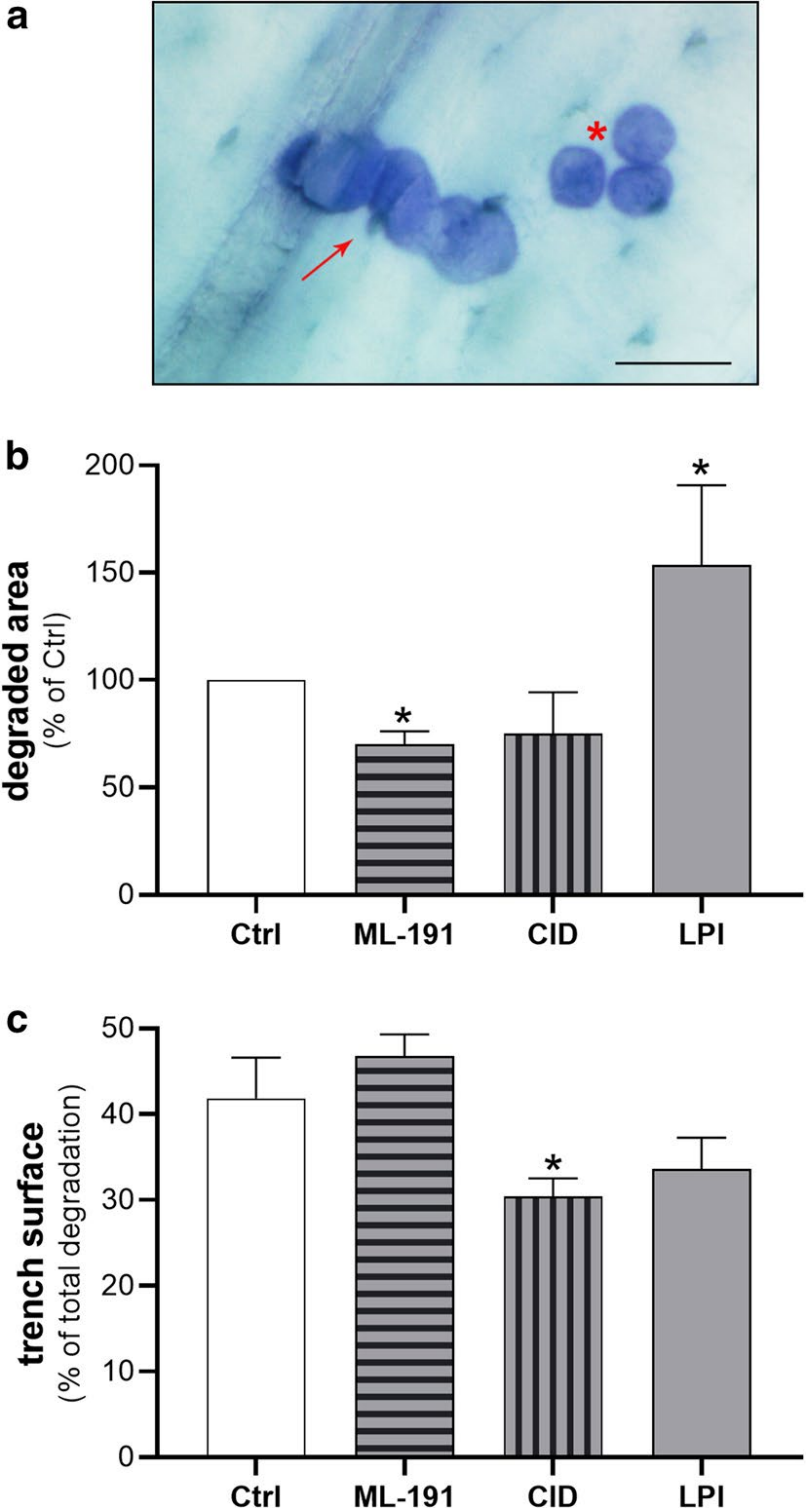
GPR55 modulators on osteoclast-resorbing activity. RAW264.7 cells were differentiated on bone slices with 15 ng/ml RANKL for 7 days in the presence of carriers or with the indicated compounds, and then the eroded surface was analysed (see “Methods”). **a** Representative image of resorption cavities, showing a trench (red arrow) and three pits (red asterisk). Scale bar, 50 µm. **b** Total resorption area on bone slice surfaces was quantified, and is here expressed as percent of the control (Ctrl) of RANKL-differentiated cells in the presence of 0.4% DMSO for the samples treated with 0.5 µM ML-191 or CID16020046, or with 0.004% fatty-acid free BSA for samples treated with 1 µM 18:0 LPI. Data are means ± SEM of at least three independent experiments performed at least in triplicate. **c** Trench cavities are expressed as the proportions (%) of the total resorption areas for each treatment. Data are means ± SEM of at least three independent experiments, performed at least in triplicate. *p < 0.05 versus Ctrl (Student’s *t*-tests). CID, CID16020046

GPR55 involvement in osteoclast functions was directly assessed using in-vitro assays of bone resorbing activity. As the transient silencing by GPR55-specific siRNAs in precursor cells was not applicable to this long-term differentiation assay, pharmacological perturbation of GPR55 was carried out with only the most specific antagonists (ML-191, CID16020046) and LPI. RAW264.7 cells were plated on cortical bone slices and RANKL-differentiated for 7 days in the absence or presence of the different GPR55 regulators. At the end of the differentiation, the cells were detached and the resorbing areas were analysed, both as total eroded surface, and as osteoclasts degradation in the pit or trench modes (see “Materials and methods” section, Fig. 11a, and [43, 56]). In contrast to the undifferentiated cells, RANKL-treated cells showed resorbing activity, with a trench surface corresponding to 42 ± 8% of total eroded surface. The total resorbing activity induced by RANKL was significantly reduced by 30% in the presence of ML-191, and 54% increased by LPI, added throughout the differentiation (see “Methods”). These compounds did not affect the relative contributions of the trenches observed with RANKL alone (Fig. 11b). The treatment with CID16020046 only slightly inhibited RANKL-promoted osteoclast degrading activity, but significantly reduced the relative trench contribution by about 30% compared to RANKL (Fig. 11c). As trenches, with respect to pit cavities, have been characterised by high erosion speed [56], the decrease in the proportion of trenches produced by CID16020046 indicated inhibition of osteoclast activity. A possible explanation for the different effects produced by the two GPR55 antagonists might be a consequence of their different actions on the receptor, as CID16020046 has been reported to act as a GPR55 inverse agonist [55].

These data support a role for GPR55 signalling in the functional resorbing activity of mature osteoclasts, even without affecting RANKL-promoted cell fusion.

## Discussion

Our research dealt with the limits of following LPI-dependent GPR55-mediated signalling under heterologous expression conditions. Using *GPR55*-silenced HeLa clones, we succeeded in comparing the signalling pathway responses of different mutant GPR55 receptors, demonstrating the requirement of lysine in position 80 of GPR55 for LPI-triggered MAPK activation and receptor internalisation. These data are in line with what was predicted by homology modelling of GPR55 with the crystal structures of the adenosine A_2A_ [45], β_2_-adrenergic [26] and δ-opioid [41] receptors, and by GPR55 docking with the LPI moiety, as no X-ray crystal structure has been reported yet for GPR55. These studies proposed the binding site for LPI, and another study for the phytocannabinoid ligands [22], set on the outer transmembrane region of GPR55, with the amino-acid residue Lys^80^ as the universal anchor, and with two closed hydrophobic regions. One of these hydrophobic pockets that is located deeper in the GPR55 binding site should accommodate the long aliphatic tail of LPI. Hydrogen bonds, van der Waals forces and hydrophobic interactions should contribute to this LPI docking to GPR55, leading to the uncovering of a G-protein binding site on the intracellular surface of the receptor, and consequently to GPR55-mediated signal transduction [45].

Receptor silencing was instrumental in the present study of the downstream effectors of the LPI/GPR55 axis in the RAW264.7 macrophages, as this allowed us to reveal the LPI-induced rise in intracellular Ca^2+^ and reorganisation of the actin cytoskeleton with filopodium formation. To the best of our knowledge, signal transduction of the GPR55 receptor has never been investigated in RAW264.7 macrophages, despite the well-characterised expression of GPR55 in several types of leukocytes (including neutrophils, lymphocytes, monocytes, macrophages) [6, 57], and its involvement in intestinal inflammation [58] and microglial-mediated neuroinflammation [59]. Increases in intracellular Ca^2+^ levels by GPR55 activation have been shown in different cellular contexts, with these triggered by both cannabinoid ligands [39], and LPIs [8, 20, 29]. Instead, the induction of filopodium formation can be accounted for by GPR55 coupling with Gα_12/13_ [20], and the consequent Cdc42 activation [60], as has been reported for cannabinoid-ligand-mediated stimulation of GPR55 [22].

RAW264.7 macrophages have been shown to express *Gpr55* mRNA at lower levels than those of another mouse macrophage cell line, the J774A.1 cells [58]. However, we observed a strong induction of *Gpr55* transcription during RANKL-induced osteoclastogenesis that was suggestive of the involvement of GPR55 in the differentiation process, or at least in mature osteoclast activity. Moreover, this increased expression of *Gpr55* mRNA levels was in line with what was observed during differentiation of primary osteoclast precursor cells from mouse bone marrow, and during human osteoclastogenesis starting with peripheral blood monocytes [9].

Indeed, GPR55 actively regulated the differentiation of RAW264.7 cells into osteoclasts, as we have shown here using both molecular and pharmacological approaches. Despite the partial reduction of *Gpr55* mRNA levels in these precursor RAW264.7 cells following *Gpr55* silencing by the siRNA treatment, this appears to be more efficient than GPR55 antagonists for the regulation of RANKL-induced osteoclastogenesis. This is indicative of the requirement for GPR55 protein expression, more than of its downstream signal transduction, as seen for the *Ctr*-transcriptional regulation. For the other differentiation marker MMP-9, while it was not affected by LPI, ML-191 reduced the RANKL-induced transcription of *Mmp-9* by only 30%, with the less potent CBD and O1918 having no effects. As *Gpr55* silencing impaired *Mmp-9* transcription more, this process would also appear to be related to *Gpr55* expression levels rather than GPR55 signalling itself.

Of note, GPR55 can form heterodimers with other receptors, and this interaction might affect their reciprocal surface expression, and in particular, their signal transduction [61]. Previous studies have demonstrated interactions between GPR55 and the cannabinoid receptor CB_2_, with both expressed and shown to have roles in bone metabolism [10, 50]. Furthermore, the cross-talk between GPR55 and the two cannabinoid receptors is further complicated through the modulation of integrin clustering [62]. Therefore, *Gpr55* silencing will not only compromised homologous signal transduction, but also result in unbalanced heterologous signal transduction, thus explaining the discrepancies between our pharmacological and molecular approaches.

RANKL-induced transcription of *Nfatc1* and *Trap* was reduced by both *Gpr55* silencing and the GPR55 antagonists, and increased by LPI, which indicates that these processes are dependent on GPR55 signal transduction. For the intermediate differentiation marker *Cathepsin-k*, both ML-191 and CBD reduced its RANKL-induced transcription, although with an expected difference in their potencies, while O1918 had no effects. LPI itself did not have any effects on *Cathepsin-k* after 72 h, while it further stimulated RANKL-induced transcription of *Cathepsin-k* at 24 h and 48 h. As RANKL-induced transcription of *Cathepsin-k* started in the first hours of the differentiation process and reached a plateau by 72 h, this might explain the lack of LPI stimulation at this later time, while indicating that this *Cathepsin-k* transcription is also dependent on GPR55 signalling.

Among the GPR55 signalling cascades, increased intracellular Ca^2+^ is one of the best candidates for modulation of RANKL-induced transcriptional remodelling in these RAW264.7 cells, as this messenger is also downstream of RANK activation by its own ligand [14], and might represent a hub for pathways that are convergent with GPR55 signal transduction. Nuclear factor-κB (NF-κB), ‘nuclear factor of activated T-cells’ (NFAT), and cAMP response element binding protein (CREB) have been identified as participants in GPR55 downstream signalling pathways in transfected HEK293 cells [63], and therefore also direct regulation of the osteoclastogenesis transcriptional processes in RAW264.7 cells by GPR55 cannot be excluded.

In the present study, the LPI/GPR55 axis induced cytoskeletal rearrangements in the RAW264.7 macrophages. Furthermore, GPR55 has been reported to regulate CB_2_-mediated chemotaxis of human neutrophils [64] and to modulate migration and polarisation of human breast cancer cells [65]. Therefore, as actin remodelling and cell migration are essential for osteoclast cell-to-cell fusion [66], these systems might explain the effects of GPR55 silencing on osteoclast syncytium formation. GPR55 agonists and antagonists were ineffective on osteoclast cell-to-cell fusion, which supports the relevance of GPR55 expression and not of its signalling in this step, and suggests a role for GPR55 interactions with and cross-regulation of other receptors, such as CB_2_ [10, 49, 50].

Altogether, the results of the present study indicate that GPR55 regulates osteoclastogenesis of RAW264.7 cells at several levels. This is favoured in terms of the transcriptional remodelling, which leads to increased levels of the main osteoclastogenesis markers. However, it is also tuned in a signalling-independent manner for the cell-to-cell fusion that is necessary for functional syncytium formation. The apparent discrepancy in GPR55 regulation is not unexpected, as although these two major steps of the differentiation process are triggered by the same stimulus (RANKL), they proceed through different signalling pathways, which are temporally regulated by various adaptor proteins, kinases and transcription factors [67]. The overall effect was an osteoclast activity dependent on GPR55 signalling, as LPI stimulates and ML-191 inhibits bone resorption, at least under the assay conditions used here.

GPR55 is overexpressed in several tumour cells [37, 68, 69], with GPR55 expression shown to correlate with tumour aggressiveness [18, 36] and GPR55 activation to promote cancer-cell proliferation both in cell culture and in xenografts in mice [68]. Therefore, we previously attempted to identify peptides that bind to human GPR55, for innovative targeting of GPR55 for potential therapeutic and/or diagnostic applications. Osteoclasts are also among the cells with the highest expression of GPR55 [9], and as GPR55 is involved in their differentiation, its targeting might be instrumental in the regulation of osteoclast activity under conditions of exacerbated bone resorption. Of these peptides that bind to human GPR55, we have shown here that peptide-P1 can recognise murine GPR55, and inhibit RAW264.7 osteoclastogenesis. The mechanism of action of this peptide is still under investigation, although we have demonstrated its intrinsic efficacy in inducing human GPR55 internalisation in a β-arrestin–independent manner, whereby this decreased plasma-membrane expression of GPR55 should account for its impaired signal transduction [38]. This previous study underlined the allosteric action of peptide-P1, as it did not compete with LPI action [38]. This is in agreement with the comparable regulation of both wild-type and GPR55-K80A mutant internalisation by peptide-P1 (data not shown), which thus indicates different anchoring requirements for peptide-P1 compared to LPI.

Peptide-P1 was also shown to inhibit GPR55-dependent proliferation of EHEB and DeFew cells, which are two human B-lymphoblastoid cell lines [38], and among other tumour cells, leukemic cells can metastasise at the bone level [70]. Bone metastasisation is a complex process that involves cross-talk between tumour cells and bone resident cells [71, 72]. Osteoclast bone resorption is essential for metastasis establishment, as upon bone degradation, growth factors are released from the bone matrix that can stimulate metastatic cell growth and survival [73]. Functional dual targeting of tumour cells and osteoclasts represents a promising target for pharmacological tools, to contain osteolytic skeletal metastasis formation, with the added potential to carry other chemotherapeutics or anti-resorptive agents. Indeed, small receptor-binding peptides have great versatility compared to other small molecules, as they can be easily functionalised for diagnostic applications or be used as carriers of other drugs, to complement their activities [74]. These peptides also have several advantages compared to monoclonal antibodies, from their simpler and more reproducible synthesis [75], to their higher penetrability and biocompatibility in vivo, along with their lower systemic toxicity that is generally consequent to their non-specific uptake into the reticulo-endothelial system [76]. The main limitation of these peptides remains their short half-life, as they can be degraded by proteases. However, the use of peptidomimetics that carry chemical modifications (e.g., cyclisation, N-terminus and C-terminus protection), or non-natural amino acids, such as D-amino acids, can overcome this limitation [77].

## Conclusions

In summary, by molecular and cell biology approaches, here, we dissected the lysophosphatidylinositol-activated GPR55-mediated signal transduction, highlighting the requirement of GPR55 Lys^80^ for lysophosphatidylinositol recognition. Moreover, we reported on a functional GPR55 in the osteoclast precursors RAW264.7 macrophages, and on the role of the lysophosphatidylinositol/GPR55 axis in the onset of their RANKL-induced osteoclastogenesis process. A phage-displayed screening of a random peptide library allowed the identification of peptide ligands of GPR55. One of the most specific, peptide-P1, was shown to be an inhibitor of this osteoclast differentiation, confirming that targeting GPR55 signalling pathway might represent a useful therapeutic option for the treatment of pathologies with exacerbated osteoclast activities. Further studies are on-going for a more complete evaluation of the pharmacological potential of peptide-P1.

## Supplementary Information

**Additional file 1**. **Table S1.** Sequences of the real-time PCR primers.

**Additional file 2**. **Table S2. **Other receptor mRNA levels under* Gpr55* silencing in RAW264.7 cells.

**Additional file 3**. **Figure S1.** LPI-induced signalling is independent of GPR55 overexpression in HEK293T and HeLa cells. (**a**) FACS analysis with an anti-HA antibody of HEK293T cells transfected with empty vector (pcDNA3) or the vector coding for GPR55 wild-type (HA-GPR55) or mutants (as indicated). (**b,** **c**) Twenty-four hours after transfection, these HEK293T cells were serum deprived for 4 h, and then stimulated with 10 µM LPI for the indicated times. Western blotting for phosphorylated (p-ERK1/2) and total ERK1/2 are shown, from a representative experiment of three independent ones. (**d**). HeLa cells transfected with empty vector (pcDNA3.1) or the vector coding for the construct ss-3×HA-GPR55 (ssGPR55). Twenty-four hours after transfection, the cells were serum deprived for 2 h, and then stimulated with 10 µM soybean LPI for the indicated times. Western blotting for phosphorylated (p-AKT, p-ERK1/2) and total AKT and ERK1/2 are shown, from a representative experiment of three independent ones.

**Additional file 4**. **Figure S2. **Osteoclastogenesis markers expression during differentiation of RAW264.7 precursors. Time-courses of *Nfatc1*, *Cathepsin-k*, *Mmp-9*, *Trap*, and *Ctr* mRNA expression levels during osteoclast differentiation of precursor RAW264.7 cells induced by 30 ng/mL RANKL. RANKL was added at time 0 and every 48 h (arrows). Transcripts were quantified by real-time PCR and normalised for *β*_*2*_*-microglobulin* expression, as the housekeeping gene. Data are means ±range from two independent experiments, and are expressed as percentages of the mRNA levels at 72 h of RANKL treatment for each marker. At this time in RANKL-treated cells compared to the undifferentiated cells *Nfatc1* was increased by 11.9 (±2.2)-fold, *Cathepsin-k* by 468.5 (±24.3)-fold, *Mmp-9* by 673.3 (±4.7)-fold, and *Trap* by 188.8 (±57.6)-fold. *Ctr* was not expressed in undifferentiated cells at any of the times analysed here. w/o, cells incubated without RANKL.

**Additional file 5**. **Figure S3.** Effects of GPR55 modulators on osteoclast maturation. (**a**, **b**) Real-time PCR analysis of the differentiation markers (as indicated) in RAW264.7 cells treated with 15 ng/mL RANKL for 72 h, in the absence or presence of 1 µM soybean LPI alone or with GPR55 antagonists (0.5 µM ML-191; 0.5 µM CID16020046). (**c**) Real-time PCR analysis of* Ctr* in RAW264.7 cells interfered with non-targeting (si-NT) or *Gpr55*-targeting (si-GPR55) siRNAs, and subsequently treated with 15 ng/mL RANKL for 72 h in the absence or presence of 1 µM soybean LPI or 1 µM ML-184. The transcripts were quantified and normalised using *β*_*2*_*-microglobulin* expression, as the housekeeping gene. Data are expressed as proportions (%) of the corresponding control RANKL, as means ±SEM from at least three independent experiments. *p <0.05, **p <0.01 (Student’s *t*-tests). RANKL, RANKL-differentiated cells. CID, CID16020046.

**Additional file 6**. **Figure S4.** Effects of GPR55 modulators on the osteoclast syncytia. RAW264.7 cells were treated with 15 ng/mL RANKL in the absence or presence of the GPR55 antagonist/agonist (1 µM ML-184, 0.5 µM CID16020046). Osteoclast syncytium formation was determined after 72 h of RANKL treatment, as number of nuclei/cell, under fluorescence microscopy. Data are means ±SE of three independent experiments. RANKL, RANKL-differentiated cells; CID, CID16020046.

**Additional file 7**. **Figure S5.** Peptide-P1 specifically binds to murine GPR55 in RAW264.7 cells. (**a**) Time course of binding of 40 µg/mL (26.8 µM) FITC-conjugated Peptide-P1 (FITC-P1) or the scrambled (KCLTSNCPK) peptide (FITC-Scr) to RAW264.7 cells at 37 °C. Peptide binding evaluated in subsequent FACS analysis of cell-associated FITC-fluorescence is shown, quantified as mean fluorescence increase compared to cells incubated in the absence of any peptide, with data representative of three independent experiments. The extrapolated apparent Kd for FITC-P1 was 22.7 µM. (b) Peptide specificity towards GPR55 was determined by incubation of 40 µg/mL FITC-labelled peptides with RAW264.7 cells treated with non-targeting (si-NT+siGLO) or* Gpr55*-targeting (si-GPR55+siGLO) siRNAs for 15 min at 37 °C. Peptide binding was subsequently evaluated by FACS analysis of cell-associated FITC fluorescence in the siGLO-positive cells, quantified as mean fluorescence increase compared to cells incubated in the absence of any peptide (see Methods). Data are means ±SEM of four independent experiments. *p <0.05 (Student’s* t*-test).

## Data Availability

The data supporting the conclusions of this article are included within the article (and its additional files) or are available from the corresponding author on reasonable request.

## Abbreviations

a.u.: Arbitrary units
BSA: Bovine serum albumin
CBD: Cannabidiol
CREB: cAMP response element binding protein
CTR: Calcitonin receptor
ERK: Extracellular signal-regulated kinase
faf: Fatty-acid-free
FITC: Fluorescein-isothiocyanate
FBS: Foetal bovine serum
GPCR: G-protein-coupled receptor
HA: Haemagglutinin
HBSS^++^: Hanks balanced salt solution with calcium and magnesium
HPRT1: Hypoxanthine phosphoribosyltransferase 1
IUPHAR: International Union of Basic and Clinical Pharmacology
LPI: Lysophosphatidylinositol
MMP-9: Matrix metalloproteinase-9
NFAT: Nuclear factor of activated T cells
Nfatc1: Nuclear factor of activated T-cells, cytoplasmic 1
NF-κB: Nuclear factor-κB
PBS: Phosphate-buffered saline
RANKL: Receptor-activator of nuclear factor kappa-β ligand
sh: Short hairpin
si: Small-interfering
ss: Signal sequence
TRAP: Tartrate-resistant acid phosphatase
