# Supplementary material for: Peptide targeting of lysophosphatidylinositol-sensing GPR55 for osteoclastogenesis tuning
Source: Cell Commun Signal. 2021 Apr 26;19:48. doi: 10.1186/s12964-021-00727-w (PMC8073907; doi:10.1186/s12964-021-00727-w)
Supplement: Supplementary file 2 — Additional file 1. Table S1. Sequences of the real-time PCR primers. [file 12964_2021_727_MOESM2_ESM.docx]

**Supplementary Table S1.** **Sequences of the real-time PCR primers.**

| **Primers** | **Annealing temperature (°C)** | **Sequence** |
| --- | --- | --- |
| Mouse *Nfatc1* | 60 | Fw: 5’-CATGCAGCCATCATCGA-3’ |
|  |  | Rv: 5’-TGGGATGTGAACTCGGAAGAC-3’ |
| Mouse *Mmp-9* | 55 | Fw: 5’-CTGTCCAGACCAAGGGTACAGCCT-3’ |
|  |  | Rv: 5’-GTGGTATAGTGGGACACATAGTGG-3’ |
| Mouse *Trap* | 55 | Fw: 5’-AAATCACTCTTTAAGACCAG-3’ |
|  |  | Rv: 5’-TTATTGAATAGCAGTGACAG-3’ |
| Mouse *Cathepsin-k* | 55 | Fw: 5’-CCTCTCTTGGTCTCCATACA-3’ |
|  |  | Rv: 5’-ATCTCTCTGTACCCTCTGCA-3’ |
| Mouse *Ctr* | 60 | Fw: 5’-ACCGACGAGCAACGCCTACGC-3’ |
|  |  | Rv: 5’-GCCTTCACAGCCTTCAGGTAC-3’ |
| Mouse *Gpr55* | 57 | Fw: 5’-TGGCCAGGCATCTTCAGT-3’ |
|  |  | Rv: 5’-CCAAGAGAAGTCCCCTTTCC-3’ |
| Mouse *Cb1 (1)* | 60 | Fw: 5’-ATCGGAGTCACCAGTGTGCTGT-3’ |
|  |  | Rv: 5’-CCTTGCCATCTTCTGAGGTGTG-3’ |
| Mouse *Cb1 (2)* | 56 | Fw: 5’-TGGCCAGGCATCTTCAGT-3’ |
|  |  | Rv: 5’-TGAAGGAGGCTGTAACCC-3’ |
| Mouse *Cb2* | 60 | Fw: 5’- TATGCTGGTTCCCTGCACTG-3’ |
|  |  | Rv: 5’-GAGCGAATCTCTCCACTCCG-3’ |
| Mouse *Lpar1* | 60 | Fw: 5’-CCAGGAGGAATCGGGACAC-3’ |
|  |  | Rv: 5’-CAATAACAAGACCAATCCCGGA-3’ |
| Mouse β*_2_-microglobulin* | 60 | Fw: 5’-TGGTGCTTGTCTCACTGACC-3’ |
|  |  | Rv: 5’-GTATGTTGGCTTCCCATTC-3’ |
| Human *GPR55* | 52 | Fw: 5’-TCTACATGATCAACCTGGCAGTCT-3’ |
|  |  | Rv: 5’-CTGGGACAGGACCATCTTGAA-3 |
| Human *HPRT1* | 60 | Fw: 5’-TGCTGACCTGCTGGATTACA-3’ |
|  |  | Rv: 5’-CCTGACCAAGGAAAGCAAAG-3’ |

Fw, forward; Rv, reverse
