# Supplementary material for: Peptide targeting of lysophosphatidylinositol-sensing GPR55 for osteoclastogenesis tuning
Source: Cell Commun Signal. 2021 Apr 26;19:48. doi: 10.1186/s12964-021-00727-w (PMC8073907; doi:10.1186/s12964-021-00727-w)
Supplement: Supplementary file 3 — Additional file 2. Table S2. Other receptor mRNA levels under Gpr55 silencing in RAW264.7 cells. [file 12964_2021_727_MOESM3_ESM.docx]

**Table S2. Other receptor mRNA levels under GPR55 silencing.**

| **Gene** | **si-GPR55**  (%si-NT) | **Control** |
| --- | --- | --- |
| *Gpr55* | 50.0 ±4.5*** |  |
| *Cb1* | ND | Mouse striatum |
| *Cb2* | 136.2 ±52.2 |  |
| *Lpar1* | 115.0 ±26.8 |  |

Receptor mRNA levels in RAW264.7 cells interfered with non-targeting (si-NT) or Gpr55-specific (si-GPR55) siRNAs were quantified by real-time PCR and normalised using *β2-microglobulin* expression, as the housekeeping gene. Data are expressed as proportion (%) of the mRNA levels in si-NT, and are means ±SEM from six independent experiments. ND, not detected (nor in si-NT cells using two different primer pairs). ****p* <0.005 (Student’s *t*-tests) *versus* si-NT.
