# Supplementary figures and images for: Peptide targeting of lysophosphatidylinositol-sensing GPR55 for osteoclastogenesis tuning
Source: Cell Commun Signal. 2021 Apr 26;19:48. doi: 10.1186/s12964-021-00727-w (PMC8073907; doi:10.1186/s12964-021-00727-w)

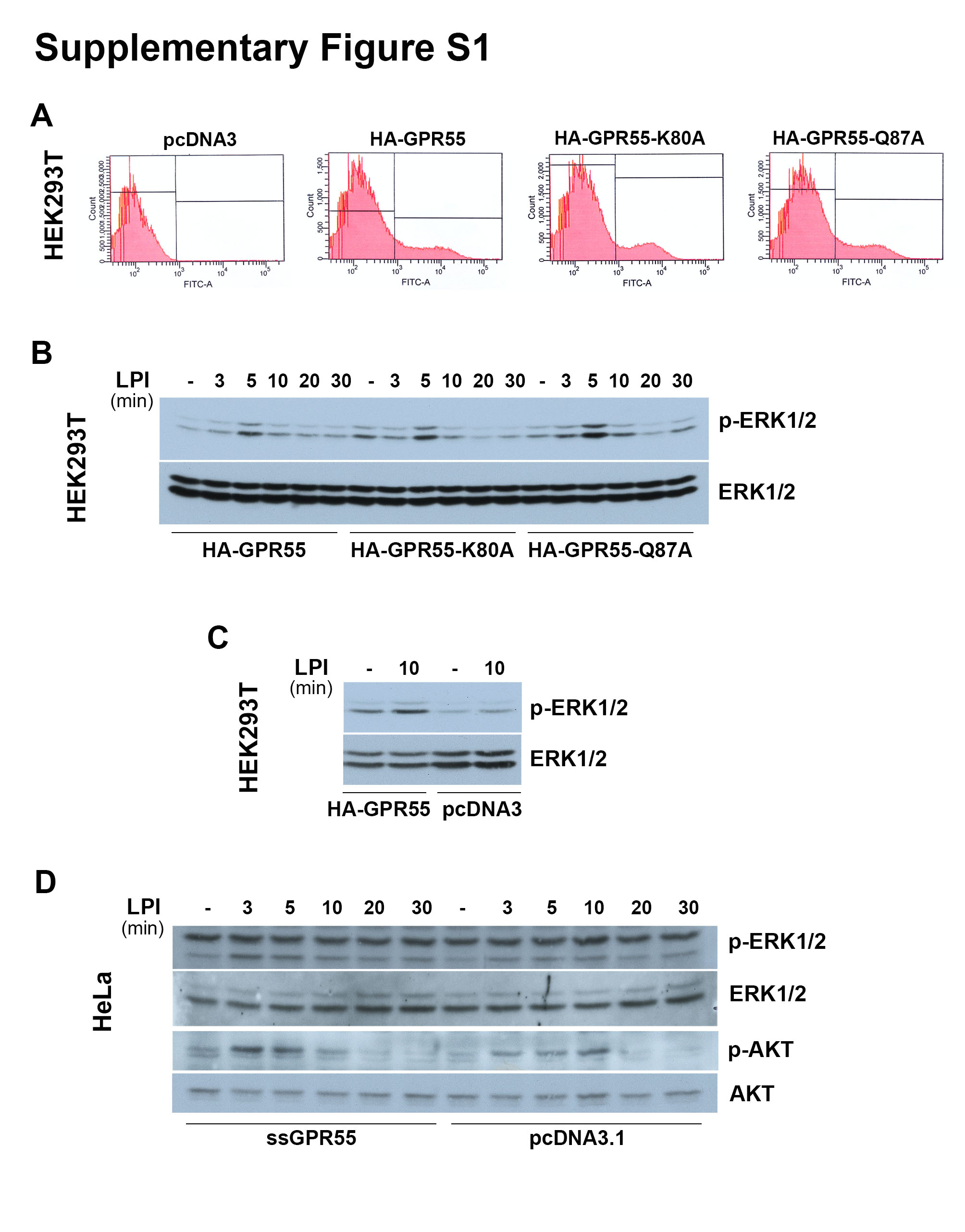

Supplement: Supplementary file 4 — Additional file 3. Figure S1. LPI-induced signalling is independent of GPR55 overexpression in HEK293T and HeLa cells. (a) FACS analysis with an anti-HA antibody of HEK293T cells transfected with empty vector (pcDNA3) or the vector coding for GPR55 wild-type (HA-GPR55) or mutants (as indicated). (b, c) Twenty-four hours after transfection, these HEK293T cells were serum deprived for 4 h, and then stimulated with 10 µM LPI for the indicated times. Western blotting for phosphorylated (p-ERK1/2) and total ERK1/2 are shown, from a representative experiment of three independent ones. (d). HeLa cells transfected with empty vector (pcDNA3.1) or the vector coding for the construct ss-3×HA-GPR55 (ssGPR55). Twenty-four hours after transfection, the cells were serum deprived for 2 h, and then stimulated with 10 µM soybean LPI for the indicated times. Western blotting for phosphorylated (p-AKT, p-ERK1/2) and total AKT and ERK1/2 are shown, from a representative experiment of three independent ones. [file 12964_2021_727_MOESM4_ESM.jpg]

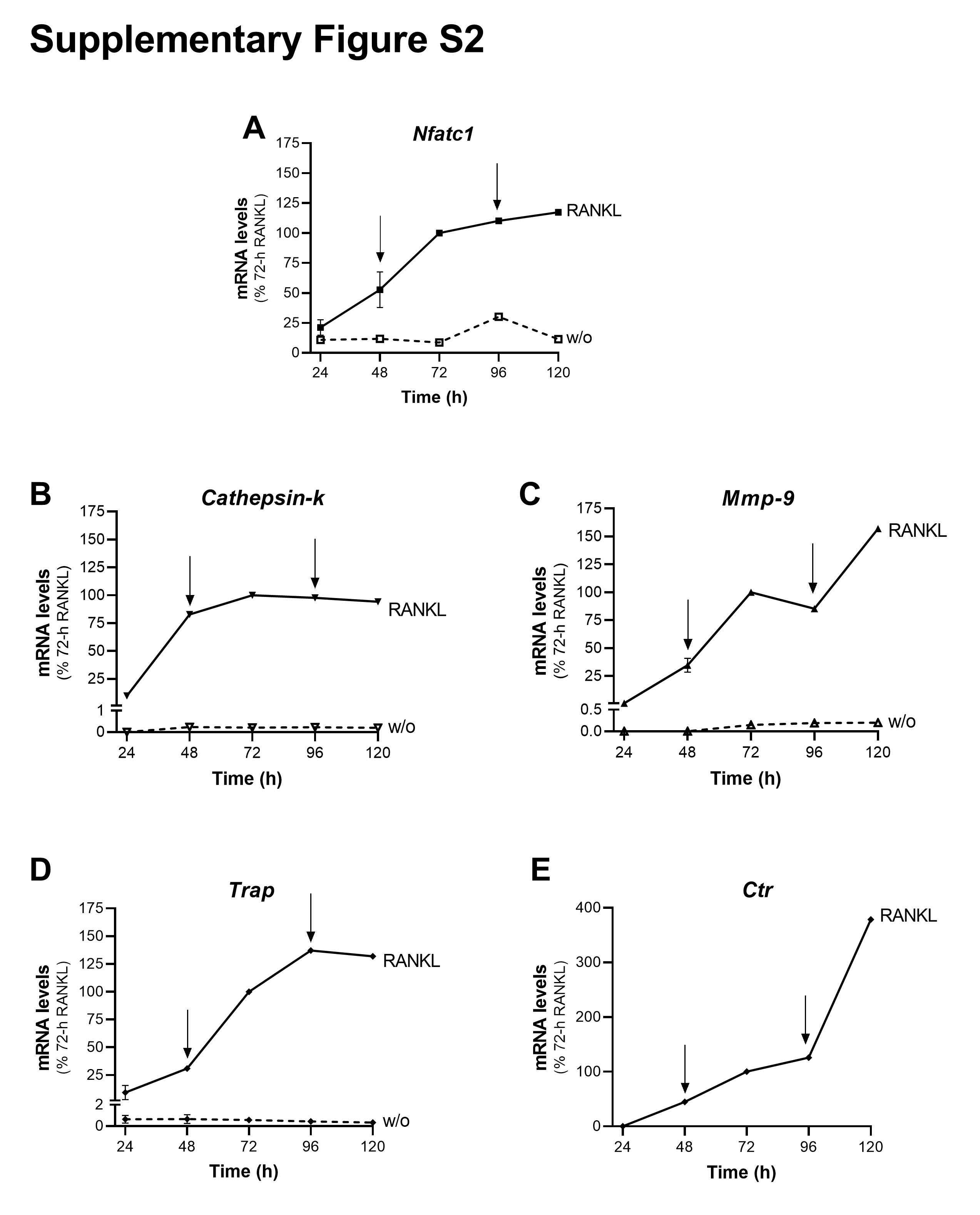

Supplement: Supplementary file 5 — Additional file 4. Figure S2. Osteoclastogenesis markers expression during differentiation of RAW264.7 precursors. Time-courses of Nfatc1, Cathepsin-k, Mmp-9, Trap, and Ctr mRNA expression levels during osteoclast differentiation of precursor RAW264.7 cells induced by 30 ng/mL RANKL. RANKL was added at time 0 and every 48 h (arrows). Transcripts were quantified by real-time PCR and normalised for β2-microglobulin expression, as the housekeeping gene. Data are means ±range from two independent experiments, and are expressed as percentages of the mRNA levels at 72 h of RANKL treatment for each marker. At this time in RANKL-treated cells compared to the undifferentiated cells Nfatc1 was increased by 11.9 (±2.2)-fold, Cathepsin-k by 468.5 (±24.3)-fold, Mmp-9 by 673.3 (±4.7)-fold, and Trap by 188.8 (±57.6)-fold. Ctr was not expressed in undifferentiated cells at any of the times analysed here. w/o, cells incubated without RANKL. [file 12964_2021_727_MOESM5_ESM.jpg]

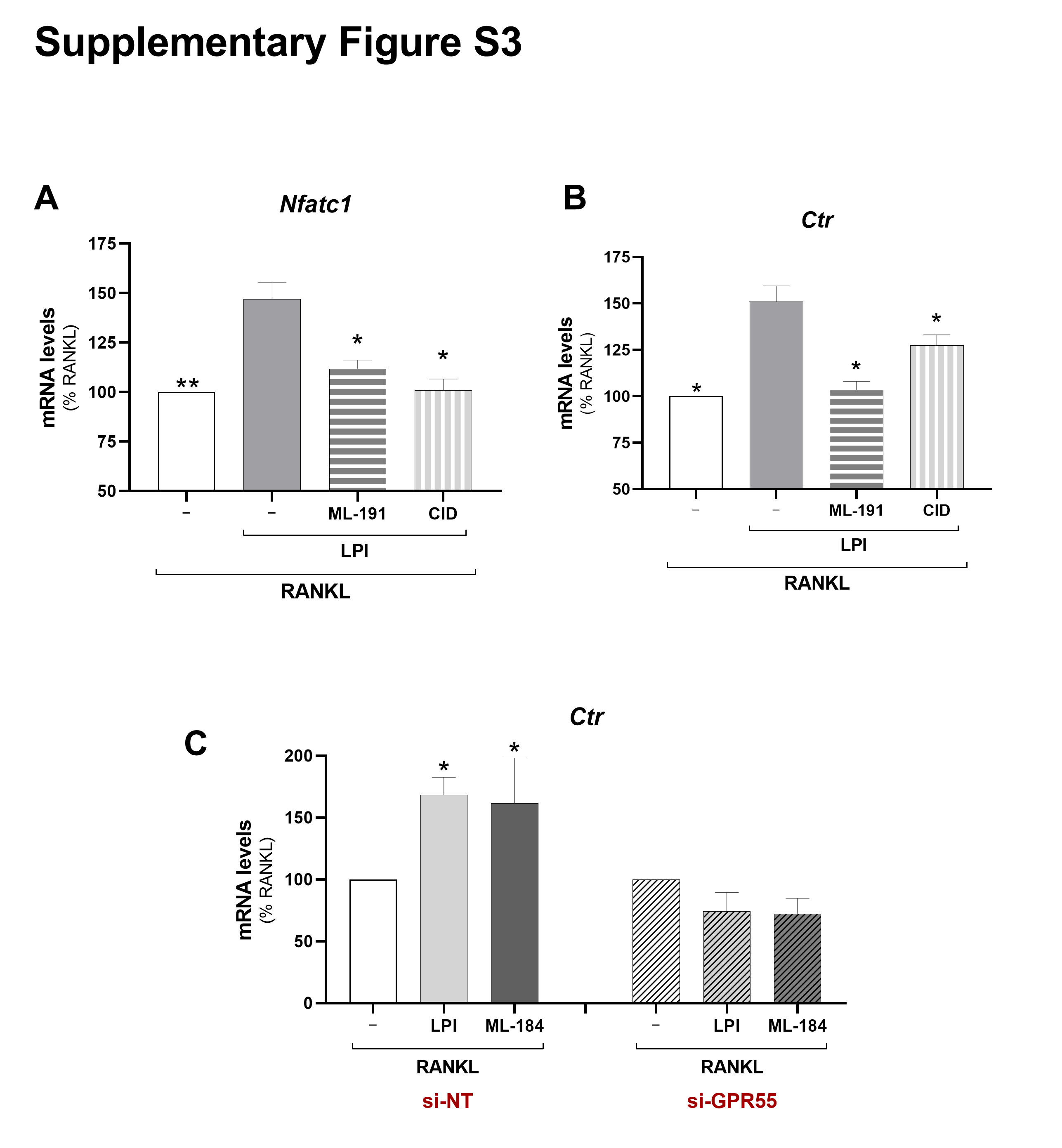

Supplement: Supplementary file 6 — Additional file 5. Figure S3. Effects of GPR55 modulators on osteoclast maturation. (a, b) Real-time PCR analysis of the differentiation markers (as indicated) in RAW264.7 cells treated with 15 ng/mL RANKL for 72 h, in the absence or presence of 1 µM soybean LPI alone or with GPR55 antagonists (0.5 µM ML-191; 0.5 µM CID16020046). (c) Real-time PCR analysis of Ctr in RAW264.7 cells interfered with non-targeting (si-NT) or Gpr55-targeting (si-GPR55) siRNAs, and subsequently treated with 15 ng/mL RANKL for 72 h in the absence or presence of 1 µM soybean LPI or 1 µM ML-184. The transcripts were quantified and normalised using β2-microglobulin expression, as the housekeeping gene. Data are expressed as proportions (%) of the corresponding control RANKL, as means ±SEM from at least three independent experiments. *p <0.05, **p <0.01 (Student’s t-tests). RANKL, RANKL-differentiated cells. CID, CID16020046. [file 12964_2021_727_MOESM6_ESM.jpg]

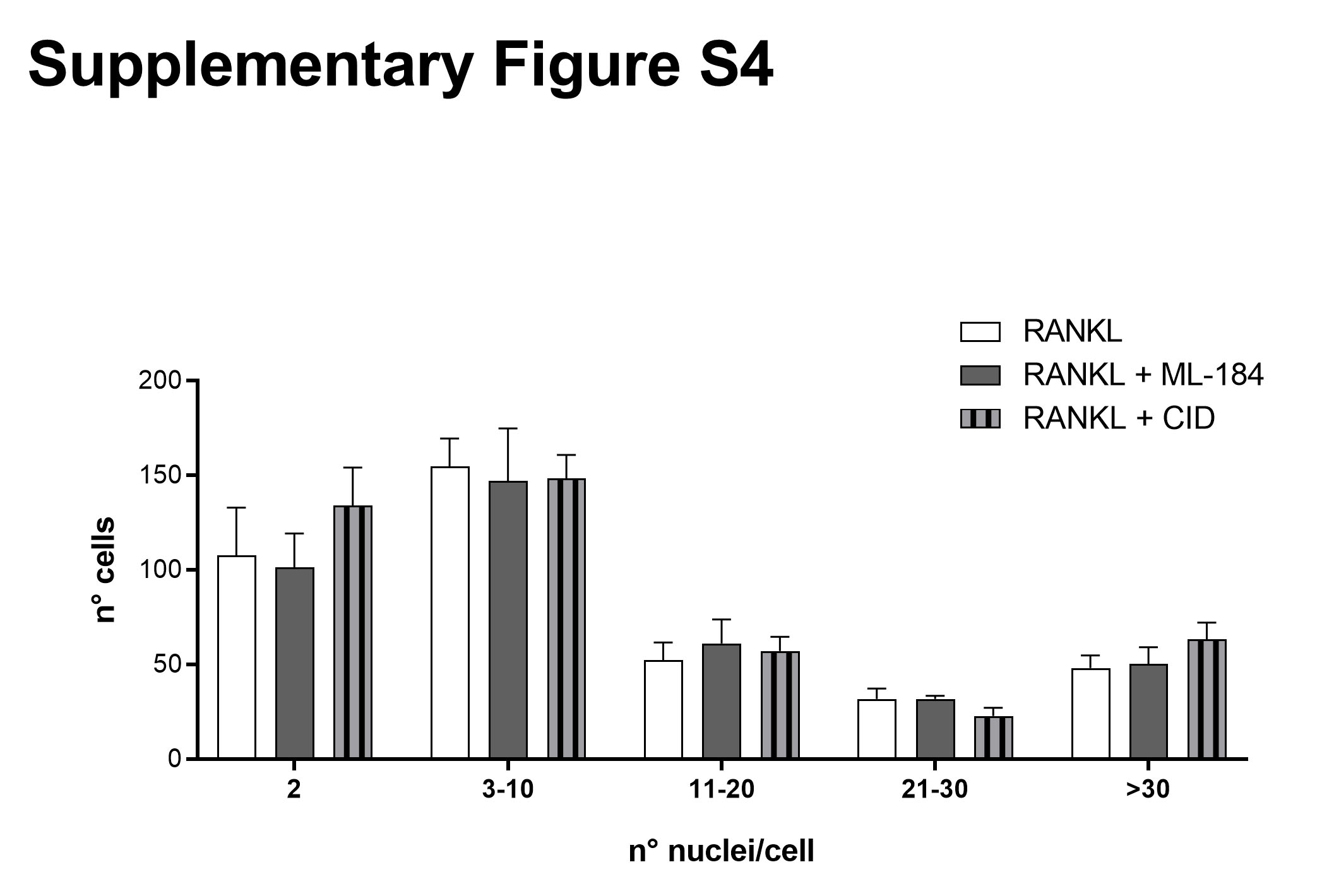

Supplement: Supplementary file 7 — Additional file 6. Figure S4. Effects of GPR55 modulators on the osteoclast syncytia. RAW264.7 cells were treated with 15 ng/mL RANKL in the absence or presence of the GPR55 antagonist/agonist (1 µM ML-184, 0.5 µM CID16020046). Osteoclast syncytium formation was determined after 72 h of RANKL treatment, as number of nuclei/cell, under fluorescence microscopy. Data are means ±SE of three independent experiments. RANKL, RANKL-differentiated cells; CID, CID16020046. [file 12964_2021_727_MOESM7_ESM.jpg]

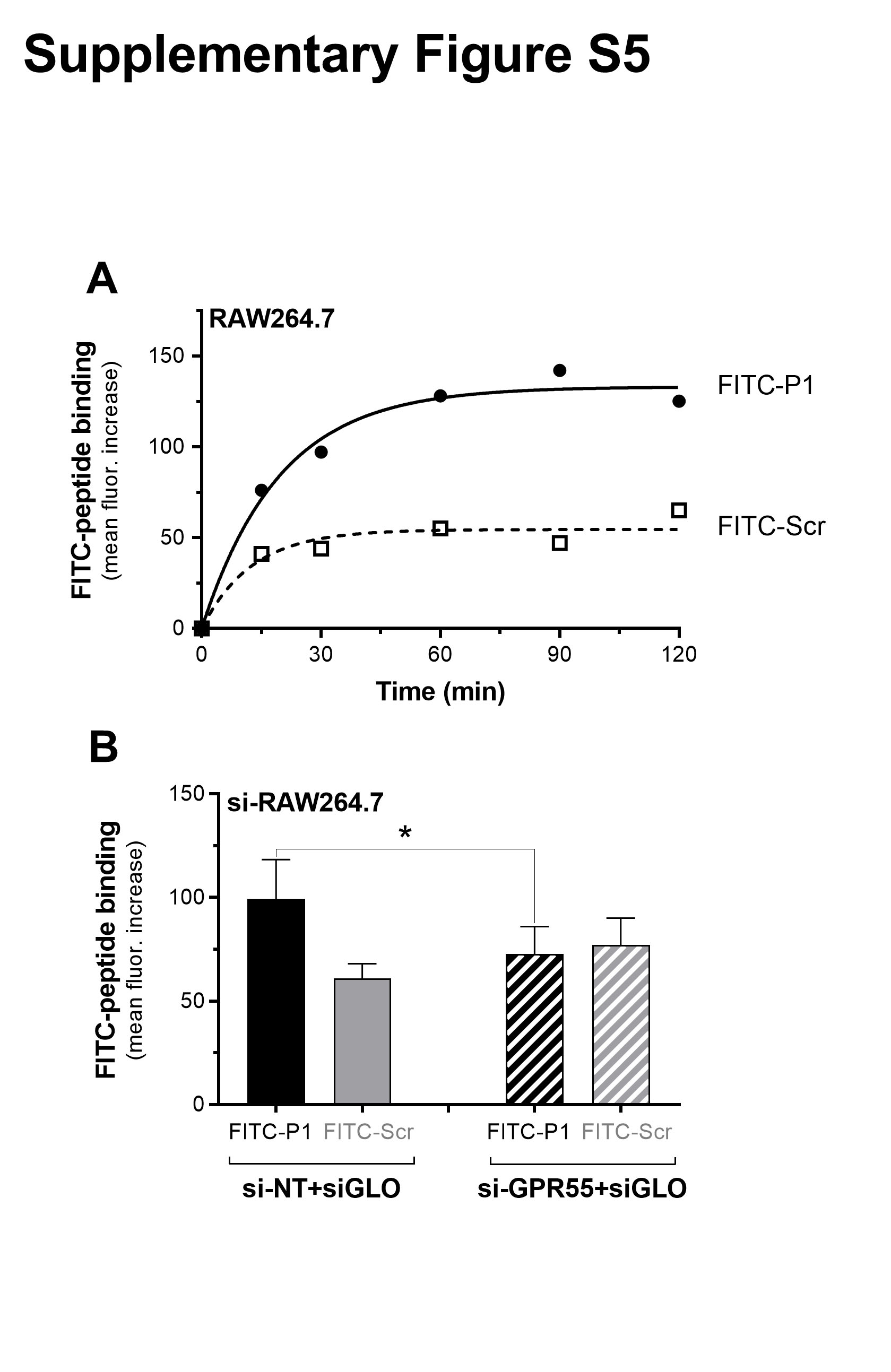

Supplement: Supplementary file 8 — Additional file 7. Figure S5. Peptide-P1 specifically binds to murine GPR55 in RAW264.7 cells. (a) Time course of binding of 40 µg/mL (26.8 µM) FITC-conjugated Peptide-P1 (FITC-P1) or the scrambled (KCLTSNCPK) peptide (FITC-Scr) to RAW264.7 cells at 37 °C. Peptide binding evaluated in subsequent FACS analysis of cell-associated FITC-fluorescence is shown, quantified as mean fluorescence increase compared to cells incubated in the absence of any peptide, with data representative of three independent experiments. The extrapolated apparent Kd for FITC-P1 was 22.7 µM. (b) Peptide specificity towards GPR55 was determined by incubation of 40 µg/mL FITC-labelled peptides with RAW264.7 cells treated with non-targeting (si-NT+siGLO) or Gpr55-targeting (si-GPR55+siGLO) siRNAs for 15 min at 37 °C. Peptide binding was subsequently evaluated by FACS analysis of cell-associated FITC fluorescence in the siGLO-positive cells, quantified as mean fluorescence increase compared to cells incubated in the absence of any peptide (see Methods). Data are means ±SEM of four independent experiments. *p <0.05 (Student’s t-test). [file 12964_2021_727_MOESM8_ESM.jpg]
